# Supplementary material for: Burden and prevalence of prognostic factors for severe COVID-19 in Sweden
Source: Eur J Epidemiol. 2020 May 18;35(5):401–9. doi: 10.1007/s10654-020-00646-z (PMC7233678; doi:10.1007/s10654-020-00646-z)
Supplement: Supplementary file 1 — Supplementary material 1 (DOCX 249 kb) [file 10654_2020_646_MOESM1_ESM.docx]

**Appendix 1.** Burden and prevalence of underlying medical conditions suggesting high risk of severe COVID-19 in each Swedish county and age group*

| County | Age groups  (years) | N | Cardiovascular disease  n (%) | Cancer  n (%) | COPD  n (%) | Severe asthma  n (%) | Diabetes  n (%) |
| --- | --- | --- | --- | --- | --- | --- | --- |
| Stockholm | 1-9 | 257368 | 1427 (0.6) | 103 (0) | 351 (0.1) | 18882 (7.3) | 381 (0.1) |
|  | 10-19 | 238146 | 2067 (0.9) | 72 (0) | 222 (0.1) | 9607 (4) | 1398 (0.6) |
|  | 20-29 | 301758 | 4222 (1.4) | 274 (0.1) | 69 (0) | 4208 (1.4) | 2556 (0.8) |
|  | 30-39 | 324475 | 6494 (2) | 743 (0.2) | 107 (0) | 4204 (1.3) | 3996 (1.2) |
|  | 40-49 | 319742 | 10447 (3.3) | 1726 (0.5) | 322 (0.1) | 4697 (1.5) | 7347 (2.3) |
|  | 50-59 | 271601 | 17106 (6.3) | 3714 (1.4) | 1478 (0.5) | 4609 (1.7) | 14704 (5.4) |
|  | 60-69 | 218741 | 28527 (13) | 7861 (3.6) | 3821 (1.7) | 4244 (1.9) | 22197 (10.1) |
|  | 70-79 | 156684 | 36414 (23.2) | 8111 (5.2) | 5815 (3.7) | 3510 (2.2) | 21924 (14) |
|  | 80+ | 85524 | 33461 (39.1) | 4630 (5.4) | 4544 (5.3) | 2290 (2.7) | 11692 (13.7) |
| Uppsala | 1-9 | 37619 | 198 (0.5) | 8 (0) | 12 (0) | 2633 (7) | 76 (0.2) |
|  | 10-19 | 37617 | 292 (0.8) | 9 (0) | 2 (0) | 1201 (3.2) | 328 (0.9) |
|  | 20-29 | 55691 | 618 (1.1) | 76 (0.1) | 8 (0) | 662 (1.2) | 589 (1.1) |
|  | 30-39 | 42875 | 735 (1.7) | 117 (0.3) | 9 (0) | 548 (1.3) | 682 (1.6) |
|  | 40-49 | 45498 | 1403 (3.1) | 287 (0.6) | 45 (0.1) | 613 (1.3) | 1210 (2.7) |
|  | 50-59 | 42280 | 2407 (5.7) | 554 (1.3) | 202 (0.5) | 673 (1.6) | 2498 (5.9) |
|  | 60-69 | 40451 | 4776 (11.8) | 1163 (2.9) | 666 (1.6) | 716 (1.8) | 4212 (10.4) |
|  | 70-79 | 28724 | 6120 (21.3) | 1346 (4.7) | 1025 (3.6) | 645 (2.2) | 4294 (14.9) |
|  | 80+ | 15570 | 5537 (35.6) | 768 (4.9) | 646 (4.1) | 502 (3.2) | 2422 (15.6) |
| Södermanland | 1-9 | 29626 | 181 (0.6) | 8 (0) | 10 (0) | 1771 (6) | 47 (0.2) |
|  | 10-19 | 31647 | 266 (0.8) | 7 (0) | 8 (0) | 951 (3) | 213 (0.7) |
|  | 20-29 | 33237 | 438 (1.3) | 19 (0.1) | 10 (0) | 371 (1.1) | 395 (1.2) |
|  | 30-39 | 30297 | 571 (1.9) | 68 (0.2) | 15 (0) | 289 (1) | 502 (1.7) |
|  | 40-49 | 36357 | 1175 (3.2) | 179 (0.5) | 65 (0.2) | 376 (1) | 1191 (3.3) |
|  | 50-59 | 35708 | 2131 (6) | 383 (1.1) | 260 (0.7) | 493 (1.4) | 2362 (6.6) |
|  | 60-69 | 36434 | 4385 (12) | 983 (2.7) | 700 (1.9) | 568 (1.6) | 3985 (10.9) |
|  | 70-79 | 28349 | 5963 (21) | 1148 (4) | 1119 (3.9) | 602 (2.1) | 4150 (14.6) |
|  | 80+ | 15565 | 5498 (35.3) | 675 (4.3) | 736 (4.7) | 440 (2.8) | 2274 (14.6) |
| Östergötland | 1-9 | 46254 | 175 (0.4) | 17 (0) | 8 (0) | 2788 (6) | 86 (0.2) |
|  | 10-19 | 47686 | 319 (0.7) | 15 (0) | 7 (0) | 1649 (3.5) | 298 (0.6) |
|  | 20-29 | 63729 | 794 (1.2) | 82 (0.1) | 17 (0) | 805 (1.3) | 719 (1.1) |
|  | 30-39 | 51592 | 1019 (2) | 138 (0.3) | 16 (0) | 611 (1.2) | 892 (1.7) |
|  | 40-49 | 57228 | 1961 (3.4) | 369 (0.6) | 66 (0.1) | 729 (1.3) | 1639 (2.9) |
|  | 50-59 | 54206 | 3477 (6.4) | 704 (1.3) | 318 (0.6) | 780 (1.4) | 3206 (5.9) |
|  | 60-69 | 52294 | 6943 (13.3) | 1593 (3) | 959 (1.8) | 884 (1.7) | 5381 (10.3) |
|  | 70-79 | 39493 | 9056 (22.9) | 2000 (5.1) | 1414 (3.6) | 889 (2.3) | 5967 (15.1) |
|  | 80+ | 23992 | 8646 (36) | 1310 (5.5) | 961 (4) | 672 (2.8) | 3741 (15.6) |
| Jönköping | 1-9 | 37064 | 140 (0.4) | 12 (0) | 32 (0.1) | 3099 (8.4) | 66 (0.2) |
|  | 10-19 | 39202 | 230 (0.6) | 10 (0) | 8 (0) | 743 (1.9) | 265 (0.7) |
|  | 20-29 | 44551 | 553 (1.2) | 52 (0.1) | 11 (0) | 523 (1.2) | 481 (1.1) |
|  | 30-39 | 38982 | 770 (2) | 116 (0.3) | 14 (0) | 372 (1) | 668 (1.7) |
|  | 40-49 | 44969 | 1680 (3.7) | 266 (0.6) | 62 (0.1) | 551 (1.2) | 1323 (2.9) |
|  | 50-59 | 42817 | 2791 (6.5) | 551 (1.3) | 253 (0.6) | 637 (1.5) | 2617 (6.1) |
|  | 60-69 | 41009 | 5410 (13.2) | 1244 (3) | 824 (2) | 760 (1.9) | 4334 (10.6) |
|  | 70-79 | 30933 | 7516 (24.3) | 1447 (4.7) | 1201 (3.9) | 740 (2.4) | 4900 (15.8) |
|  | 80+ | 19917 | 7904 (39.7) | 1040 (5.2) | 890 (4.5) | 696 (3.5) | 3374 (16.9) |
| Kronoberg | 1-9 | 20056 | 71 (0.4) | 9 (0) | 11 (0.1) | 1908 (9.5) | 32 (0.2) |
|  | 10-19 | 20851 | 144 (0.7) | 6 (0) | 7 (0) | 1221 (5.9) | 154 (0.7) |
|  | 20-29 | 25096 | 307 (1.2) | 20 (0.1) | 3 (0) | 278 (1.1) | 306 (1.2) |
|  | 30-39 | 21767 | 393 (1.8) | 43 (0.2) | 1 (0) | 185 (0.8) | 425 (2) |
|  | 40-49 | 24043 | 745 (3.1) | 138 (0.6) | 27 (0.1) | 205 (0.9) | 630 (2.6) |
|  | 50-59 | 22751 | 1327 (5.8) | 298 (1.3) | 110 (0.5) | 225 (1) | 1251 (5.5) |
|  | 60-69 | 23156 | 2768 (12) | 733 (3.2) | 417 (1.8) | 205 (0.9) | 2266 (9.8) |
|  | 70-79 | 17275 | 3744 (21.7) | 793 (4.6) | 634 (3.7) | 212 (1.2) | 2518 (14.6) |
|  | 80+ | 11379 | 3984 (35) | 600 (5.3) | 494 (4.3) | 169 (1.5) | 1690 (14.9) |
| Kalmar | 1-9 | 22045 | 137 (0.6) | 12 (0.1) | 5 (0) | 1677 (7.6) | 43 (0.2) |
|  | 10-19 | 24031 | 184 (0.8) | 2 (0) | 1 (0) | 843 (3.5) | 155 (0.6) |
|  | 20-29 | 28573 | 356 (1.2) | 27 (0.1) | 6 (0) | 275 (1) | 285 (1) |
|  | 30-39 | 23800 | 469 (2) | 75 (0.3) | 5 (0) | 183 (0.8) | 354 (1.5) |
|  | 40-49 | 29294 | 966 (3.3) | 175 (0.6) | 42 (0.1) | 299 (1) | 724 (2.5) |
|  | 50-59 | 30664 | 1970 (6.4) | 360 (1.2) | 197 (0.6) | 360 (1.2) | 1795 (5.9) |
|  | 60-69 | 32677 | 4324 (13.2) | 1013 (3.1) | 585 (1.8) | 428 (1.3) | 3499 (10.7) |
|  | 70-79 | 25606 | 6101 (23.8) | 1178 (4.6) | 939 (3.7) | 419 (1.6) | 4092 (16) |
|  | 80+ | 15266 | 5763 (37.8) | 728 (4.8) | 668 (4.4) | 298 (2) | 2400 (15.7) |
| Gotland | 1-9 | 5251 | 23 (0.4) | 0 (0) | 3 (0.1) | 447 (8.5) | 12 (0.2) |
|  | 10-19 | 5721 | 44 (0.8) | 1 (0) | 1 (0) | 289 (5.1) | 44 (0.8) |
|  | 20-29 | 6871 | 116 (1.7) | 6 (0.1) | 0 (0) | 85 (1.2) | 95 (1.4) |
|  | 30-39 | 5476 | 104 (1.9) | 12 (0.2) | 4 (0.1) | 54 (1) | 120 (2.2) |
|  | 40-49 | 7250 | 261 (3.6) | 48 (0.7) | 10 (0.1) | 49 (0.7) | 184 (2.5) |
|  | 50-59 | 7961 | 518 (6.5) | 87 (1.1) | 43 (0.5) | 70 (0.9) | 368 (4.6) |
|  | 60-69 | 8366 | 1054 (12.6) | 245 (2.9) | 144 (1.7) | 93 (1.1) | 816 (9.8) |
|  | 70-79 | 6288 | 1436 (22.8) | 300 (4.8) | 218 (3.5) | 86 (1.4) | 891 (14.2) |
|  | 80+ | 3455 | 1253 (36.3) | 193 (5.6) | 126 (3.6) | 46 (1.3) | 492 (14.2) |
| Blekinge | 1-9 | 15176 | 57 (0.4) | 7 (0) | 3 (0) | 918 (6) | 32 (0.2) |
|  | 10-19 | 16381 | 114 (0.7) | 6 (0) | 3 (0) | 576 (3.5) | 127 (0.8) |
|  | 20-29 | 18602 | 228 (1.2) | 16 (0.1) | 5 (0) | 251 (1.3) | 221 (1.2) |
|  | 30-39 | 16639 | 362 (2.2) | 48 (0.3) | 8 (0) | 163 (1) | 315 (1.9) |
|  | 40-49 | 19941 | 690 (3.5) | 105 (0.5) | 36 (0.2) | 246 (1.2) | 556 (2.8) |
|  | 50-59 | 19340 | 1233 (6.4) | 253 (1.3) | 116 (0.6) | 261 (1.3) | 1046 (5.4) |
|  | 60-69 | 19965 | 2544 (12.7) | 629 (3.2) | 369 (1.8) | 304 (1.5) | 2025 (10.1) |
|  | 70-79 | 16453 | 3670 (22.3) | 761 (4.6) | 530 (3.2) | 300 (1.8) | 2352 (14.3) |
|  | 80+ | 9639 | 3491 (36.2) | 533 (5.5) | 365 (3.8) | 238 (2.5) | 1406 (14.6) |
| Skåne | 1-9 | 141556 | 685 (0.5) | 62 (0) | 77 (0.1) | 9305 (6.6) | 240 (0.2) |
|  | 10-19 | 136630 | 1176 (0.9) | 43 (0) | 22 (0) | 5478 (4) | 934 (0.7) |
|  | 20-29 | 174415 | 2399 (1.4) | 161 (0.1) | 44 (0) | 2030 (1.2) | 1895 (1.1) |
|  | 30-39 | 164660 | 3506 (2.1) | 415 (0.3) | 59 (0) | 1705 (1) | 3005 (1.8) |
|  | 40-49 | 171851 | 6160 (3.6) | 1083 (0.6) | 259 (0.2) | 2072 (1.2) | 4722 (2.7) |
|  | 50-59 | 157164 | 10982 (7) | 2130 (1.4) | 1019 (0.6) | 1936 (1.2) | 9620 (6.1) |
|  | 60-69 | 147565 | 20793 (14.1) | 4688 (3.2) | 2846 (1.9) | 2142 (1.5) | 16697 (11.3) |
|  | 70-79 | 112083 | 27786 (24.8) | 5751 (5.1) | 4465 (4) | 1966 (1.8) | 17538 (15.6) |
|  | 80+ | 66387 | 25432 (38.3) | 3511 (5.3) | 3000 (4.5) | 1330 (2) | 9608 (14.5) |
| Halland | 1-9 | 33766 | 174 (0.5) | 8 (0) | 12 (0) | 2249 (6.7) | 52 (0.2) |
|  | 10-19 | 35312 | 301 (0.9) | 8 (0) | 4 (0) | 1746 (4.9) | 268 (0.8) |
|  | 20-29 | 37421 | 465 (1.2) | 29 (0.1) | 3 (0) | 497 (1.3) | 410 (1.1) |
|  | 30-39 | 34016 | 682 (2) | 115 (0.3) | 9 (0) | 354 (1) | 502 (1.5) |
|  | 40-49 | 42196 | 1282 (3) | 294 (0.7) | 41 (0.1) | 496 (1.2) | 922 (2.2) |
|  | 50-59 | 40052 | 2360 (5.9) | 579 (1.4) | 149 (0.4) | 470 (1.2) | 1843 (4.6) |
|  | 60-69 | 38614 | 4906 (12.7) | 1295 (3.4) | 515 (1.3) | 486 (1.3) | 3384 (8.8) |
|  | 70-79 | 29724 | 6887 (23.2) | 1622 (5.5) | 893 (3) | 549 (1.8) | 3955 (13.3) |
|  | 80+ | 17475 | 6562 (37.6) | 1117 (6.4) | 650 (3.7) | 453 (2.6) | 2317 (13.3) |
| Västra Götaland | 1-9 | 174363 | 923 (0.5) | 87 (0) | 67 (0) | 9927 (5.7) | 321 (0.2) |
|  | 10-19 | 173804 | 1340 (0.8) | 52 (0) | 19 (0) | 5065 (2.9) | 1253 (0.7) |
|  | 20-29 | 227114 | 2714 (1.2) | 221 (0.1) | 38 (0) | 1965 (0.9) | 2119 (0.9) |
|  | 30-39 | 205385 | 3792 (1.8) | 552 (0.3) | 67 (0) | 1651 (0.8) | 2727 (1.3) |
|  | 40-49 | 216672 | 6821 (3.1) | 1298 (0.6) | 243 (0.1) | 1935 (0.9) | 5628 (2.6) |
|  | 50-59 | 204787 | 12265 (6) | 2650 (1.3) | 978 (0.5) | 2112 (1) | 11388 (5.6) |
|  | 60-69 | 188492 | 23010 (12.2) | 5528 (2.9) | 2736 (1.5) | 2411 (1.3) | 19615 (10.4) |
|  | 70-79 | 137907 | 30886 (22.4) | 6495 (4.7) | 4341 (3.1) | 2320 (1.7) | 20438 (14.8) |
|  | 80+ | 83660 | 30169 (36.1) | 4627 (5.5) | 3056 (3.7) | 1967 (2.4) | 11863 (14.2) |
| Värmland | 1-9 | 25711 | 126 (0.5) | 10 (0) | 3 (0) | 1877 (7.3) | 53 (0.2) |
|  | 10-19 | 28202 | 195 (0.7) | 12 (0) | 1 (0) | 1218 (4.3) | 185 (0.7) |
|  | 20-29 | 34737 | 405 (1.2) | 30 (0.1) | 4 (0) | 420 (1.2) | 386 (1.1) |
|  | 30-39 | 28276 | 510 (1.8) | 78 (0.3) | 8 (0) | 317 (1.1) | 512 (1.8) |
|  | 40-49 | 34905 | 1113 (3.2) | 204 (0.6) | 27 (0.1) | 415 (1.2) | 1100 (3.2) |
|  | 50-59 | 36072 | 2302 (6.4) | 446 (1.2) | 170 (0.5) | 492 (1.4) | 2237 (6.2) |
|  | 60-69 | 36579 | 4827 (13.2) | 1056 (2.9) | 552 (1.5) | 498 (1.4) | 4461 (12.2) |
|  | 70-79 | 28020 | 6655 (23.8) | 1260 (4.5) | 835 (3) | 472 (1.7) | 4902 (17.5) |
|  | 80+ | 17626 | 6560 (37.2) | 863 (4.9) | 589 (3.3) | 339 (1.9) | 2948 (16.7) |
| Örebro | 1-9 | 30190 | 132 (0.4) | 11 (0) | 6 (0) | 1742 (5.8) | 48 (0.2) |
|  | 10-19 | 31488 | 199 (0.6) | 7 (0) | 6 (0) | 953 (3) | 226 (0.7) |
|  | 20-29 | 38926 | 432 (1.1) | 37 (0.1) | 5 (0) | 343 (0.9) | 395 (1) |
|  | 30-39 | 32700 | 606 (1.9) | 76 (0.2) | 8 (0) | 253 (0.8) | 578 (1.8) |
|  | 40-49 | 37379 | 1240 (3.3) | 212 (0.6) | 44 (0.1) | 350 (0.9) | 1102 (2.9) |
|  | 50-59 | 35295 | 2172 (6.2) | 354 (1) | 180 (0.5) | 384 (1.1) | 2189 (6.2) |
|  | 60-69 | 35638 | 4510 (12.7) | 963 (2.7) | 552 (1.5) | 436 (1.2) | 3836 (10.8) |
|  | 70-79 | 27424 | 5779 (21.1) | 1145 (4.2) | 726 (2.6) | 450 (1.6) | 4224 (15.4) |
|  | 80+ | 15534 | 5274 (34) | 758 (4.9) | 512 (3.3) | 332 (2.1) | 2233 (14.4) |
| Västmanland | 1-9 | 26914 | 213 (0.8) | 16 (0.1) | 9 (0) | 1687 (6.3) | 56 (0.2) |
|  | 10-19 | 28327 | 228 (0.8) | 4 (0) | 3 (0) | 1022 (3.6) | 238 (0.8) |
|  | 20-29 | 33453 | 416 (1.2) | 25 (0.1) | 5 (0) | 302 (0.9) | 373 (1.1) |
|  | 30-39 | 29465 | 644 (2.2) | 79 (0.3) | 10 (0) | 267 (0.9) | 528 (1.8) |
|  | 40-49 | 34302 | 1171 (3.4) | 177 (0.5) | 47 (0.1) | 361 (1.1) | 1093 (3.2) |
|  | 50-59 | 33375 | 2190 (6.6) | 365 (1.1) | 174 (0.5) | 398 (1.2) | 2134 (6.4) |
|  | 60-69 | 32287 | 4480 (13.9) | 917 (2.8) | 589 (1.8) | 486 (1.5) | 3637 (11.3) |
|  | 70-79 | 25477 | 6217 (24.4) | 1210 (4.7) | 881 (3.5) | 491 (1.9) | 3975 (15.6) |
|  | 80+ | 14858 | 5555 (37.4) | 803 (5.4) | 526 (3.5) | 432 (2.9) | 2206 (14.8) |
| Dalarna | 1-9 | 27171 | 108 (0.4) | 9 (0) | 4 (0) | 1973 (7.3) | 50 (0.2) |
|  | 10-19 | 29705 | 192 (0.6) | 8 (0) | 2 (0) | 1772 (6) | 187 (0.6) |
|  | 20-29 | 32842 | 443 (1.3) | 25 (0.1) | 1 (0) | 388 (1.2) | 410 (1.2) |
|  | 30-39 | 28896 | 602 (2.1) | 72 (0.2) | 6 (0) | 240 (0.8) | 603 (2.1) |
|  | 40-49 | 34436 | 1226 (3.6) | 168 (0.5) | 47 (0.1) | 346 (1) | 1107 (3.2) |
|  | 50-59 | 36400 | 2491 (6.8) | 381 (1) | 158 (0.4) | 376 (1) | 2155 (5.9) |
|  | 60-69 | 38984 | 5371 (13.8) | 989 (2.5) | 514 (1.3) | 478 (1.2) | 4298 (11) |
|  | 70-79 | 29040 | 7133 (24.6) | 1114 (3.8) | 832 (2.9) | 522 (1.8) | 4666 (16.1) |
|  | 80+ | 17396 | 6639 (38.2) | 767 (4.4) | 569 (3.3) | 417 (2.4) | 2807 (16.1) |
| Gävleborg | 1-9 | 26806 | 93 (0.3) | 8 (0) | 3 (0) | 1946 (7.3) | 56 (0.2) |
|  | 10-19 | 29791 | 170 (0.6) | 8 (0) | 7 (0) | 1151 (3.9) | 230 (0.8) |
|  | 20-29 | 34042 | 482 (1.4) | 28 (0.1) | 3 (0) | 387 (1.1) | 420 (1.2) |
|  | 30-39 | 28416 | 612 (2.2) | 69 (0.2) | 4 (0) | 318 (1.1) | 473 (1.7) |
|  | 40-49 | 36062 | 1205 (3.3) | 196 (0.5) | 34 (0.1) | 335 (0.9) | 1087 (3) |
|  | 50-59 | 36573 | 2476 (6.8) | 391 (1.1) | 166 (0.5) | 450 (1.2) | 2253 (6.2) |
|  | 60-69 | 38056 | 5086 (13.4) | 972 (2.6) | 554 (1.5) | 568 (1.5) | 4341 (11.4) |
|  | 70-79 | 29101 | 7055 (24.2) | 1385 (4.8) | 880 (3) | 704 (2.4) | 4577 (15.7) |
|  | 80+ | 16704 | 6393 (38.3) | 804 (4.8) | 648 (3.9) | 494 (3) | 2594 (15.5) |
| Västernorrland | 1-9 | 23341 | 97 (0.4) | 5 (0) | 8 (0) | 1580 (6.8) | 56 (0.2) |
|  | 10-19 | 25872 | 192 (0.7) | 6 (0) | 2 (0) | 1094 (4.2) | 200 (0.8) |
|  | 20-29 | 28126 | 358 (1.3) | 21 (0.1) | 6 (0) | 269 (1) | 342 (1.2) |
|  | 30-39 | 25584 | 484 (1.9) | 61 (0.2) | 2 (0) | 197 (0.8) | 470 (1.8) |
|  | 40-49 | 31346 | 944 (3) | 168 (0.5) | 22 (0.1) | 246 (0.8) | 998 (3.2) |
|  | 50-59 | 31343 | 2035 (6.5) | 350 (1.1) | 131 (0.4) | 336 (1.1) | 2014 (6.4) |
|  | 60-69 | 32820 | 4233 (12.9) | 861 (2.6) | 436 (1.3) | 368 (1.1) | 3973 (12.1) |
|  | 70-79 | 25536 | 5917 (23.2) | 1077 (4.2) | 662 (2.6) | 403 (1.6) | 4141 (16.2) |
|  | 80+ | 14804 | 5232 (35.3) | 610 (4.1) | 425 (2.9) | 271 (1.8) | 2351 (15.9) |
| Jämtland | 1-9 | 12444 | 49 (0.4) | 5 (0) | 3 (0) | 574 (4.6) | 21 (0.2) |
|  | 10-19 | 13035 | 94 (0.7) | 5 (0) | 0 (0) | 368 (2.8) | 108 (0.8) |
|  | 20-29 | 15756 | 167 (1.1) | 8 (0.1) | 6 (0) | 254 (1.6) | 151 (1) |
|  | 30-39 | 13427 | 219 (1.6) | 40 (0.3) | 2 (0) | 187 (1.4) | 202 (1.5) |
|  | 40-49 | 15887 | 438 (2.8) | 82 (0.5) | 17 (0.1) | 195 (1.2) | 379 (2.4) |
|  | 50-59 | 16409 | 898 (5.5) | 211 (1.3) | 66 (0.4) | 221 (1.3) | 948 (5.8) |
|  | 60-69 | 17355 | 1962 (11.3) | 479 (2.8) | 249 (1.4) | 249 (1.4) | 1977 (11.4) |
|  | 70-79 | 12653 | 2596 (20.5) | 567 (4.5) | 379 (3) | 249 (2) | 2081 (16.4) |
|  | 80+ | 7720 | 2469 (32) | 320 (4.1) | 297 (3.8) | 188 (2.4) | 1287 (16.7) |
| Västerbotten | 1-9 | 26267 | 161 (0.6) | 13 (0) | 20 (0.1) | 1738 (6.6) | 45 (0.2) |
|  | 10-19 | 27265 | 219 (0.8) | 9 (0) | 4 (0) | 881 (3.2) | 224 (0.8) |
|  | 20-29 | 39437 | 455 (1.2) | 32 (0.1) | 12 (0) | 460 (1.2) | 391 (1) |
|  | 30-39 | 30837 | 469 (1.5) | 70 (0.2) | 13 (0) | 311 (1) | 473 (1.5) |
|  | 40-49 | 32146 | 958 (3) | 161 (0.5) | 36 (0.1) | 405 (1.3) | 846 (2.6) |
|  | 50-59 | 31955 | 1934 (6.1) | 378 (1.2) | 107 (0.3) | 475 (1.5) | 1823 (5.7) |
|  | 60-69 | 32344 | 4173 (12.9) | 850 (2.6) | 384 (1.2) | 533 (1.6) | 3298 (10.2) |
|  | 70-79 | 23829 | 5618 (23.6) | 999 (4.2) | 678 (2.8) | 600 (2.5) | 3435 (14.4) |
|  | 80+ | 14291 | 5350 (37.4) | 586 (4.1) | 454 (3.2) | 416 (2.9) | 2140 (15) |
| Norrbotten | 1-9 | 22481 | 71 (0.3) | 6 (0) | 5 (0) | 1828 (8.1) | 44 (0.2) |
|  | 10-19 | 25524 | 165 (0.6) | 8 (0) | 1 (0) | 1282 (5) | 157 (0.6) |
|  | 20-29 | 32506 | 321 (1) | 16 (0) | 10 (0) | 359 (1.1) | 325 (1) |
|  | 30-39 | 25488 | 420 (1.6) | 57 (0.2) | 4 (0) | 224 (0.9) | 380 (1.5) |
|  | 40-49 | 31251 | 916 (2.9) | 123 (0.4) | 33 (0.1) | 345 (1.1) | 823 (2.6) |
|  | 50-59 | 33550 | 2185 (6.5) | 326 (1) | 154 (0.5) | 523 (1.6) | 1976 (5.9) |
|  | 60-69 | 34194 | 4606 (13.5) | 796 (2.3) | 511 (1.5) | 664 (1.9) | 3669 (10.7) |
|  | 70-79 | 25619 | 6476 (25.3) | 940 (3.7) | 854 (3.3) | 665 (2.6) | 4117 (16.1) |
|  | 80+ | 14727 | 5629 (38.2) | 518 (3.5) | 543 (3.7) | 517 (3.5) | 2414 (16.4) |

*On the 1^st^ of January 2016. Prevalence measures calculated for a three-years look back period.

**Appendix 2.** Burden and prevalence of prognostic factors for severe COVID-19 for different look back periods*

| Underlying medical condition | 1-year | 5-years | 10-years |
| --- | --- | --- | --- |
| Cardiovascular disease (%) | 389774 (4.0) | 928436 (9.6) | 1233745 (12.8) |
| Cancer (%) | 50037 (0.5) | 190294 (2.0) | 299388 (3.1) |
| COPD (%) | 46529 (0.5) | 95662 (1.0) | 115688 (1.2) |
| Severe asthma (%) | 108458 (1.1) | 292624 (3.0) | 424554 (4.4) |
| Diabetes (%) | 439127 (4.6) | 489095 (5.1) | 513335 (5.3) |
| Number of prognostic factors* |  |  |  |
| At least one (%) | 1864867 (19.4) | 2335829 (24.3) | 2652601 (27.6) |
| At least two (%) | 435492 (4.5) | 778925 (8.1) | 955883 (9.9) |
| At least three (%) | 80993 (0.8) | 203152 (2.1) | 278659 (2.9) |

COPD: Chronic obstructive pulmonary disease

* Prognostic factors: >70 years of age, cardiovascular disease, cancer, COPD, severe asthma or diabetes

**Appendix 3.** Burden and prevalence of underlying medical conditions for severe COVID-19 in each Swedish county for different look back periods*

| County | Overall,  N | Look-back period,  (in years) | Cardiovascular disease,  n (%) | Cancer,  n (%) | COPD,  n (%) | Severe asthma,  n (%) | Diabetes,  n (%) |
| --- | --- | --- | --- | --- | --- | --- | --- |
| Stockholm | 2174039 | 1 | 79976 (3.7) | 9688 (0.4) | 9718 (0.4) | 27497 (1.3) | 79366 (3.7) |
|  |  | 3 | 140165 (6.4) | 27234 (1.3) | 16729 (0.8) | 56251 (2.6) | 86195 (4.0) |
|  |  | 5 | 184025 (8.5) | 39498 (1.8) | 20263 (0.9) | 75310 (3.5) | 89783 (4.1) |
|  |  | 10 | 246578 (11.3) | 61677 (2.8) | 24511 (1.1) | 108338 (5) | 95457 (4.4) |
| Uppsala | 346325 | 1 | 12256 (3.5) | 1714 (0.5) | 1601 (0.5) | 4459 (1.3) | 15187 (4.4) |
|  |  | 3 | 22086 (6.4) | 4328 (1.2) | 2615 (0.8) | 8193 (2.4) | 16311 (4.7) |
|  |  | 5 | 29116 (8.4) | 6300 (1.8) | 3178 (0.9) | 10394 (3) | 16887 (4.9) |
|  |  | 10 | 38853 (11.2) | 9741 (2.8) | 3782 (1.1) | 14587 (4.2) | 17733 (5.1) |
| Södermanland | 277220 | 1 | 10842 (3.9) | 1284 (0.5) | 1692 (0.6) | 2710 (1) | 14247 (5.1) |
|  |  | 3 | 20608 (7.4) | 3470 (1.3) | 2923 (1.1) | 5861 (2.1) | 15119 (5.5) |
|  |  | 5 | 27620 (10) | 5137 (1.9) | 3595 (1.3) | 8171 (2.9) | 15663 (5.7) |
|  |  | 10 | 36265 (13.1) | 8322 (3) | 4181 (1.5) | 11789 (4.3) | 16230 (5.9) |
| Östergötland | 436474 | 1 | 17703 (4.1) | 2496 (0.6) | 2310 (0.5) | 5343 (1.2) | 20528 (4.7) |
|  |  | 3 | 32390 (7.4) | 6228 (1.4) | 3766 (0.9) | 9807 (2.2) | 21929 (5) |
|  |  | 5 | 42180 (9.7) | 9250 (2.1) | 4487 (1) | 12887 (3) | 22656 (5.2) |
|  |  | 10 | 55758 (12.8) | 14228 (3.3) | 5299 (1.2) | 18652 (4.3) | 23724 (5.4) |
| Jönköping | 339444 | 1 | 15299 (4.5) | 1889 (0.6) | 2034 (0.6) | 4331 (1.3) | 16929 (5) |
|  |  | 3 | 26994 (8) | 4738 (1.4) | 3295 (1) | 8121 (2.4) | 18028 (5.3) |
|  |  | 5 | 34578 (10.2) | 6979 (2.1) | 3770 (1.1) | 10366 (3.1) | 18608 (5.5) |
|  |  | 10 | 45293 (13.3) | 11081 (3.3) | 4369 (1.3) | 13546 (4) | 19441 (5.7) |
| Kronoberg | 186374 | 1 | 7179 (3.9) | 1057 (0.6) | 1074 (0.6) | 2400 (1.3) | 8590 (4.6) |
|  |  | 3 | 13483 (7.2) | 2640 (1.4) | 1704 (0.9) | 4608 (2.5) | 9272 (5) |
|  |  | 5 | 18007 (9.7) | 3966 (2.1) | 2075 (1.1) | 6531 (3.5) | 9629 (5.2) |
|  |  | 10 | 24158 (13) | 6400 (3.4) | 2433 (1.3) | 9173 (4.9) | 10094 (5.4) |
| Kalmar | 231956 | 1 | 10835 (4.7) | 1284 (0.6) | 1453 (0.6) | 2516 (1.1) | 12438 (5.4) |
|  |  | 3 | 20270 (8.7) | 3570 (1.5) | 2448 (1.1) | 4782 (2.1) | 13347 (5.8) |
|  |  | 5 | 26275 (11.3) | 5302 (2.3) | 2984 (1.3) | 6298 (2.7) | 13815 (6) |
|  |  | 10 | 34963 (15.1) | 8526 (3.7) | 3477 (1.5) | 8675 (3.7) | 14405 (6.2) |
| Gotland | 56639 | 1 | 2695 (4.8) | 356 (0.6) | 338 (0.6) | 650 (1.1) | 2860 (5) |
|  |  | 3 | 4809 (8.5) | 892 (1.6) | 549 (1) | 1219 (2.2) | 3022 (5.3) |
|  |  | 5 | 6373 (11.3) | 1261 (2.2) | 647 (1.1) | 1678 (3) | 3101 (5.5) |
|  |  | 10 | 8445 (14.9) | 1972 (3.5) | 784 (1.4) | 2475 (4.4) | 3246 (5.7) |
| Blekinge | 152136 | 1 | 6565 (4.3) | 952 (0.6) | 895 (0.6) | 1603 (1.1) | 7601 (5) |
|  |  | 3 | 12389 (8.1) | 2358 (1.5) | 1435 (0.9) | 3257 (2.1) | 8080 (5.3) |
|  |  | 5 | 16507 (10.9) | 3565 (2.3) | 1748 (1.1) | 4496 (3) | 8875 (5.8) |
|  |  | 10 | 22252 (14.6) | 5595 (3.7) | 2105 (1.4) | 6459 (4.2) | 9229 (6.1) |
| Skåne | 1272311 | 1 | 54139 (4.3) | 7185 (0.6) | 6995 (0.5) | 13072 (1) | 59021 (4.6) |
|  |  | 3 | 98919 (7.8) | 17844 (1.4) | 11791 (0.9) | 27964 (2.2) | 64259 (5.1) |
|  |  | 5 | 130942 (10.3) | 26201 (2.1) | 14421 (1.1) | 39106 (3.1) | 67391 (5.3) |
|  |  | 10 | 171208 (13.5) | 41205 (3.2) | 17792 (1.4) | 58297 (4.6) | 71504 (5.6) |
| Halland | 308576 | 1 | 12050 (3.9) | 1918 (0.6) | 1323 (0.4) | 3961 (1.3) | 12860 (4.2) |
|  |  | 3 | 23619 (7.7) | 5067 (1.6) | 2276 (0.7) | 7300 (2.4) | 13653 (4.4) |
|  |  | 5 | 31590 (10.2) | 7665 (2.5) | 2842 (0.9) | 9882 (3.2) | 14086 (4.6) |
|  |  | 10 | 42549 (13.8) | 11752 (3.8) | 3571 (1.2) | 14672 (4.8) | 14775 (4.8) |
| Västra Götaland | 1612184 | 1 | 60292 (3.7) | 8428 (0.5) | 6735 (0.4) | 14844 (0.9) | 70745 (4.4) |
|  |  | 3 | 111920 (6.9) | 21510 (1.3) | 11545 (0.7) | 29353 (1.8) | 75352 (4.7) |
|  |  | 5 | 147274 (9.1) | 31900 (2) | 14238 (0.9) | 39380 (2.4) | 77992 (4.8) |
|  |  | 10 | 198323 (12.3) | 50479 (3.1) | 17500 (1.1) | 58700 (3.6) | 81581 (5.1) |
| Värmland | 270128 | 1 | 12159 (4.5) | 1655 (0.6) | 1243 (0.5) | 2937 (1.1) | 15715 (5.8) |
|  |  | 3 | 22693 (8.4) | 3959 (1.5) | 2189 (0.8) | 6048 (2.2) | 16784 (6.2) |
|  |  | 5 | 28942 (10.7) | 5785 (2.1) | 2630 (1) | 8156 (3) | 17358 (6.4) |
|  |  | 10 | 36913 (13.7) | 8965 (3.3) | 3028 (1.1) | 10484 (3.9) | 18025 (6.7) |
| Örebro | 284574 | 1 | 10683 (3.8) | 1568 (0.6) | 1202 (0.4) | 2507 (0.9) | 13976 (4.9) |
|  |  | 3 | 20344 (7.1) | 3563 (1.3) | 2039 (0.7) | 5243 (1.8) | 14831 (5.2) |
|  |  | 5 | 27266 (9.6) | 5174 (1.8) | 2459 (0.9) | 7388 (2.6) | 15283 (5.4) |
|  |  | 10 | 35856 (12.6) | 8335 (2.9) | 3017 (1.1) | 10714 (3.8) | 15780 (5.5) |
| Västmanland | 258458 | 1 | 11879 (4.6) | 1428 (0.6) | 1338 (0.5) | 2692 (1) | 13367 (5.2) |
|  |  | 3 | 21114 (8.2) | 3596 (1.4) | 2244 (0.9) | 5446 (2.1) | 14240 (5.5) |
|  |  | 5 | 26967 (10.4) | 5308 (2.1) | 2648 (1) | 7381 (2.9) | 14641 (5.7) |
|  |  | 10 | 35856 (12.6) | 8335 (2.9) | 3017 (1.1) | 10714 (3.8) | 15780 (5.5) |
| Dalarna | 274870 | 1 | 14061 (5.1) | 1366 (0.5) | 1303 (0.5) | 3354 (1.2) | 15291 (5.6) |
|  |  | 3 | 24205 (8.8) | 3533 (1.3) | 2133 (0.8) | 6512 (2.4) | 16283 (5.9) |
|  |  | 5 | 30872 (11.2) | 5378 (2) | 2662 (1) | 9132 (3.3) | 16824 (6.1) |
|  |  | 10 | 35990 (13.9) | 8371 (3.2) | 3199 (1.2) | 10738 (4.2) | 15113 (5.8) |
| Gävleborg | 275551 | 1 | 14045 (5.1) | 1617 (0.6) | 1413 (0.5) | 3508 (1.3) | 15024 (5.5) |
|  |  | 3 | 23572 (8.6) | 3861 (1.4) | 2299 (0.8) | 6353 (2.3) | 16031 (5.8) |
|  |  | 5 | 29981 (10.9) | 5528 (2) | 2724 (1) | 8168 (3) | 16587 (6) |
|  |  | 10 | 41222 (15) | 8588 (3.1) | 3238 (1.2) | 13938 (5.1) | 17434 (6.3) |
| Västernorrland | 238772 | 1 | 10618 (4.4) | 1211 (0.5) | 1027 (0.4) | 2600 (1.1) | 13781 (5.8) |
|  |  | 3 | 19492 (8.2) | 3159 (1.3) | 1694 (0.7) | 4764 (2) | 14545 (6.1) |
|  |  | 5 | 25234 (10.6) | 4612 (1.9) | 2090 (0.9) | 6560 (2.7) | 15027 (6.3) |
|  |  | 10 | 39496 (14.3) | 8499 (3.1) | 3141 (1.1) | 11571 (4.2) | 17234 (6.3) |
| Jämtland | 124686 | 1 | 4390 (3.5) | 595 (0.5) | 576 (0.5) | 1251 (1) | 6721 (5.4) |
|  |  | 3 | 8892 (7.1) | 1717 (1.4) | 1019 (0.8) | 2485 (2) | 7154 (5.7) |
|  |  | 5 | 11573 (9.3) | 2579 (2.1) | 1260 (1) | 3304 (2.6) | 7350 (5.9) |
|  |  | 10 | 33225 (13.9) | 7316 (3.1) | 2493 (1) | 9756 (4.1) | 15655 (6.6) |
| Västerbotten | 258371 | 1 | 10178 (3.9) | 1226 (0.5) | 957 (0.4) | 2860 (1.1) | 11944 (4.6) |
|  |  | 3 | 19337 (7.5) | 3098 (1.2) | 1708 (0.7) | 5819 (2.3) | 12675 (4.9) |
|  |  | 5 | 25542 (9.9) | 4697 (1.8) | 2129 (0.8) | 8676 (3.4) | 13072 (5.1) |
|  |  | 10 | 34132 (13.2) | 7594 (2.9) | 2690 (1) | 13693 (5.3) | 13563 (5.2) |
| Norrbotten | 245340 | 1 | 11930 (4.9) | 1120 (0.5) | 1302 (0.5) | 3363 (1.4) | 12936 (5.3) |
|  |  | 3 | 20789 (8.5) | 2790 (1.1) | 2115 (0.9) | 6407 (2.6) | 13905 (5.7) |
|  |  | 5 | 27572 (11.2) | 4209 (1.7) | 2812 (1.1) | 9360 (3.8) | 14467 (5.9) |
|  |  | 10 | 36907 (15) | 6684 (2.7) | 3557 (1.4) | 13550 (5.5) | 15564 (6.3) |

COPD: Chronic obstructive pulmonary disease. *Baseline measure is on the 1^st^ of January 2016.

**Appendix 4**. Burden and prevalence of underlying medical conditions suggesting high risk for severe COVID-19 in each age group for different look back periods*

| Age groups  (years) | Look-back period  (in years) | Cardiovascular disease, n (%) | Cancer,  n (%) | COPD,  n (%) | Severe asthma,  n (%) | Diabetes,  n (%) |
| --- | --- | --- | --- | --- | --- | --- |
| 1-9 | 1 | 2553 (0.2) | 135 (0.0) | 306 (0.0) | 40118 (3.9) | 1774 (0.2) |
|  | 5 | 7246 (0.7) | 599 (0.1) | 834 (0.1) | 89578 (8.6) | 1873 (0.2) |
|  | 10 | 8990 (0.9) | 841 (0.1) | 1010 (0.1) | 107982 (10.4) | 1919 (0.2) |
| 10-19 | 1 | 4006 (0.4) | 112 (0.0) | 185 (0.0) | 20766 (2.0) | 6952 (0.7) |
|  | 5 | 11524 (1.1) | 475 (0.0) | 419 (0.0) | 52546 (5.0) | 7329 (0.7) |
|  | 10 | 17554 (1.7) | 883 (0.1) | 616 (0.1) | 87777 (8.4) | 7505 (0.7) |
| 20-29 | 1 | 7276 (0.6) | 458 (0.0) | 113 (0.0) | 5858 (0.4) | 11851 (0.9) |
|  | 5 | 25799 (2.0) | 1699 (0.1) | 429 (0.0) | 24976 (1.9) | 14154 (1.1) |
|  | 10 | 39441 (3.0) | 2435 (0.2) | 686 (0.1) | 51010 (3.9) | 14963 (1.1) |
| 30-39 | 1 | 10317 (0.9) | 1173 (0.1) | 178 (0.0) | 5369 (0.4) | 15264 (1.3) |
|  | 5 | 37430 (3.1) | 4428 (0.4) | 543 (0.0) | 18309 (1.5) | 20778 (1.7) |
|  | 10 | 57378 (4.8) | 6542 (0.5) | 843 (0.1) | 27840 (2.3) | 24271 (2.0) |
| 40-49 | 1 | 20695 (1.6) | 2848 (0.2) | 779 (0.1) | 6778 (0.5) | 31753 (2.4) |
|  | 5 | 65423 (5.0) | 10859 (0.8) | 2024 (0.2) | 21539 (1.7) | 36919 (2.8) |
|  | 10 | 100270 (7.7) | 16098 (1.2) | 2812 (0.2) | 31637 (2.4) | 41606 (3.2) |
| 50-59 | 1 | 40732 (3.3) | 5958 (0.5) | 3503 (0.3) | 7387 (0.6) | 66576 (5.5) |
|  | 5 | 108147 (8.9) | 22687 (1.9) | 8154 (0.7) | 22530 (1.8) | 72705 (6.0) |
|  | 10 | 155905 (12.8) | 34273 (2.8) | 10502 (0.9) | 32206 (2.6) | 75567 (6.2) |
| 60-69 | 1 | 82237 (7.2) | 13520 (1.2) | 10984 (1.0) | 8180 (0.7) | 116184 (10.1) |
|  | 5 | 195046 (17) | 50598 (4.4) | 23453 (2.0) | 23833 (2.1) | 124914 (10.9) |
|  | 10 | 261615 (22.8) | 76699 (6.7) | 28625 (2.5) | 33229 (2.9) | 128620 (11.2) |
| 70-79 | 1 | 114983 (13.4) | 15702 (1.8) | 17935 (2.1) | 7941 (0.9) | 121822 (14.2) |
|  | 5 | 249939 (29.2) | 60526 (7.1) | 35183 (4.1) | 22734 (2.7) | 132664 (15.5) |
|  | 10 | 317353 (37.1) | 98201 (11.5) | 41656 (4.9) | 31005 (3.6) | 136990 (16.0) |
| 80+ | 1 | 106975 (21.3) | 10131 (2.0) | 12546 (2.5) | 6061 (1.2) | 66951 (13.4) |
|  | 5 | 227882 (45.4) | 38423 (7.7) | 24623 (4.9) | 16579 (3.3) | 77759 (15.5) |
|  | 10 | 275239 (54.9) | 63416 (12.6) | 28938 (5.8) | 21868 (4.4) | 81894 (16.3) |

COPD: Chronic obstructive pulmonary disease. *Baseline measure is on 1^st^ of January 2016.

**Appendix 6.** Burden and prevalence of underlying medical conditions suggesting high-risk of severe COVID-19 risk factors by each Swedish county and age groups for a one-year look-back period.*

| County | Age groups  (years) | N | Cardiovascular disease  n (%) | Cancer  n (%) | COPD  n (%) | Severe asthma  n (%) | Diabetes  n (%) |
| --- | --- | --- | --- | --- | --- | --- | --- |
| Stockholm | 1-9 | 257368 | 750 (0.3) | 35 (0) | 211 (0.1) | 10299 (4) | 367 (0.1) |
|  | 10-19 | 238146 | 1090 (0.5) | 24 (0) | 129 (0.1) | 4997 (2.1) | 1344 (0.6) |
|  | 20-29 | 301758 | 1992 (0.7) | 97 (0) | 22 (0) | 1730 (0.6) | 2332 (0.8) |
|  | 30-39 | 324475 | 3109 (1) | 259 (0.1) | 42 (0) | 1816 (0.6) | 3357 (1) |
|  | 40-49 | 319742 | 5216 (1.6) | 646 (0.2) | 151 (0) | 2006 (0.6) | 6642 (2.1) |
|  | 50-59 | 271601 | 9370 (3.4) | 1361 (0.5) | 762 (0.3) | 2023 (0.7) | 13752 (5.1) |
|  | 60-69 | 218741 | 16299 (7.5) | 2568 (1.2) | 2154 (1) | 1905 (0.9) | 20861 (9.5) |
|  | 70-79 | 156684 | 21971 (14) | 2911 (1.9) | 3478 (2.2) | 1640 (1) | 20355 (13) |
|  | 80+ | 85524 | 20179 (23.6) | 1787 (2.1) | 2769 (3.2) | 1081 (1.3) | 10356 (12.1) |
| Uppsala | 1-9 | 37619 | 86 (0.2) | 7 (0) | 4 (0) | 1715 (4.6) | 74 (0.2) |
|  | 10-19 | 37617 | 139 (0.4) | 5 (0) | 1 (0) | 729 (1.9) | 300 (0.8) |
|  | 20-29 | 55691 | 265 (0.5) | 37 (0.1) | 4 (0) | 259 (0.5) | 525 (0.9) |
|  | 30-39 | 42875 | 311 (0.7) | 41 (0.1) | 7 (0) | 249 (0.6) | 575 (1.3) |
|  | 40-49 | 45498 | 709 (1.6) | 114 (0.3) | 20 (0) | 274 (0.6) | 1090 (2.4) |
|  | 50-59 | 42280 | 1243 (2.9) | 208 (0.5) | 127 (0.3) | 354 (0.8) | 2363 (5.6) |
|  | 60-69 | 40451 | 2734 (6.8) | 465 (1.1) | 397 (1) | 337 (0.8) | 4012 (9.9) |
|  | 70-79 | 28724 | 3625 (12.6) | 534 (1.9) | 656 (2.3) | 311 (1.1) | 4075 (14.2) |
|  | 80+ | 15570 | 3144 (20.2) | 303 (1.9) | 385 (2.5) | 231 (1.5) | 2173 (14) |
| Södermanland | 1-9 | 29626 | 81 (0.3) | 3 (0) | 5 (0) | 941 (3.2) | 47 (0.2) |
|  | 10-19 | 31647 | 130 (0.4) | 2 (0) | 2 (0) | 494 (1.6) | 212 (0.7) |
|  | 20-29 | 33237 | 161 (0.5) | 11 (0) | 5 (0) | 117 (0.4) | 357 (1.1) |
|  | 30-39 | 30297 | 208 (0.7) | 27 (0.1) | 7 (0) | 109 (0.4) | 432 (1.4) |
|  | 40-49 | 36357 | 572 (1.6) | 64 (0.2) | 36 (0.1) | 152 (0.4) | 1118 (3.1) |
|  | 50-59 | 35708 | 1082 (3) | 150 (0.4) | 140 (0.4) | 202 (0.6) | 2238 (6.3) |
|  | 60-69 | 36434 | 2311 (6.3) | 378 (1) | 396 (1.1) | 236 (0.6) | 3816 (10.5) |
|  | 70-79 | 28349 | 3241 (11.4) | 415 (1.5) | 658 (2.3) | 250 (0.9) | 3936 (13.9) |
|  | 80+ | 15565 | 3056 (19.6) | 234 (1.5) | 443 (2.8) | 209 (1.3) | 2091 (13.4) |
| Östergötland | 1-9 | 46254 | 95 (0.2) | 5 (0) | 4 (0) | 1711 (3.7) | 86 (0.2) |
|  | 10-19 | 47686 | 165 (0.3) | 4 (0) | 7 (0) | 931 (2) | 295 (0.6) |
|  | 20-29 | 63729 | 337 (0.5) | 33 (0.1) | 10 (0) | 374 (0.6) | 652 (1) |
|  | 30-39 | 51592 | 478 (0.9) | 55 (0.1) | 10 (0) | 292 (0.6) | 727 (1.4) |
|  | 40-49 | 57228 | 954 (1.7) | 150 (0.3) | 40 (0.1) | 360 (0.6) | 1522 (2.7) |
|  | 50-59 | 54206 | 1838 (3.4) | 294 (0.5) | 180 (0.3) | 386 (0.7) | 3066 (5.7) |
|  | 60-69 | 52294 | 3861 (7.4) | 658 (1.3) | 596 (1.1) | 465 (0.9) | 5156 (9.9) |
|  | 70-79 | 39493 | 5154 (13.1) | 756 (1.9) | 882 (2.2) | 476 (1.2) | 5648 (14.3) |
|  | 80+ | 23992 | 4821 (20.1) | 541 (2.3) | 581 (2.4) | 348 (1.5) | 3376 (14.1) |
| Jönköping | 1-9 | 37064 | 74 (0.2) | 6 (0) | 8 (0) | 2049 (5.5) | 65 (0.2) |
|  | 10-19 | 39202 | 130 (0.3) | 8 (0) | 6 (0) | 343 (0.9) | 264 (0.7) |
|  | 20-29 | 44551 | 267 (0.6) | 22 (0) | 4 (0) | 207 (0.5) | 430 (1) |
|  | 30-39 | 38982 | 373 (1) | 37 (0.1) | 9 (0) | 155 (0.4) | 566 (1.5) |
|  | 40-49 | 44969 | 843 (1.9) | 114 (0.3) | 26 (0.1) | 217 (0.5) | 1243 (2.8) |
|  | 50-59 | 42817 | 1530 (3.6) | 217 (0.5) | 150 (0.4) | 260 (0.6) | 2489 (5.8) |
|  | 60-69 | 41009 | 3029 (7.4) | 513 (1.3) | 509 (1.2) | 349 (0.9) | 4162 (10.1) |
|  | 70-79 | 30933 | 4406 (14.2) | 558 (1.8) | 759 (2.5) | 388 (1.3) | 4642 (15) |
|  | 80+ | 19917 | 4647 (23.3) | 414 (2.1) | 563 (2.8) | 363 (1.8) | 3068 (15.4) |
| Kronoberg | 1-9 | 20056 | 40 (0.2) | 1 (0) | 8 (0) | 1037 (5.2) | 32 (0.2) |
|  | 10-19 | 20851 | 66 (0.3) | 4 (0) | 3 (0) | 662 (3.2) | 154 (0.7) |
|  | 20-29 | 25096 | 124 (0.5) | 13 (0.1) | 0 (0) | 92 (0.4) | 277 (1.1) |
|  | 30-39 | 21767 | 165 (0.8) | 21 (0.1) | 0 (0) | 97 (0.4) | 354 (1.6) |
|  | 40-49 | 24043 | 350 (1.5) | 52 (0.2) | 13 (0.1) | 99 (0.4) | 589 (2.4) |
|  | 50-59 | 22751 | 643 (2.8) | 119 (0.5) | 62 (0.3) | 120 (0.5) | 1189 (5.2) |
|  | 60-69 | 23156 | 1496 (6.5) | 288 (1.2) | 255 (1.1) | 105 (0.5) | 2152 (9.3) |
|  | 70-79 | 17275 | 2040 (11.8) | 313 (1.8) | 413 (2.4) | 101 (0.6) | 2348 (13.6) |
|  | 80+ | 11379 | 2255 (19.8) | 246 (2.2) | 320 (2.8) | 87 (0.8) | 1495 (13.1) |
| Kalmar | 1-9 | 22045 | 56 (0.3) | 4 (0) | 0 (0) | 1004 (4.6) | 42 (0.2) |
|  | 10-19 | 24031 | 86 (0.4) | 2 (0) | 0 (0) | 463 (1.9) | 155 (0.6) |
|  | 20-29 | 28573 | 138 (0.5) | 9 (0) | 5 (0) | 115 (0.4) | 264 (0.9) |
|  | 30-39 | 23800 | 197 (0.8) | 28 (0.1) | 2 (0) | 63 (0.3) | 314 (1.3) |
|  | 40-49 | 29294 | 458 (1.6) | 63 (0.2) | 17 (0.1) | 142 (0.5) | 666 (2.3) |
|  | 50-59 | 30664 | 976 (3.2) | 120 (0.4) | 108 (0.4) | 161 (0.5) | 1705 (5.6) |
|  | 60-69 | 32677 | 2304 (7.1) | 373 (1.1) | 316 (1) | 216 (0.7) | 3339 (10.2) |
|  | 70-79 | 25606 | 3344 (13.1) | 426 (1.7) | 602 (2.4) | 197 (0.8) | 3828 (14.9) |
|  | 80+ | 15266 | 3276 (21.5) | 259 (1.7) | 403 (2.6) | 155 (1) | 2125 (13.9) |
| Gotland | 1-9 | 5251 | 11 (0.2) | 0 (0) | 1 (0) | 261 (5) | 12 (0.2) |
|  | 10-19 | 5721 | 20 (0.3) | 1 (0) | 1 (0) | 177 (3.1) | 44 (0.8) |
|  | 20-29 | 6871 | 56 (0.8) | 1 (0) | 0 (0) | 37 (0.5) | 88 (1.3) |
|  | 30-39 | 5476 | 54 (1) | 5 (0.1) | 1 (0) | 22 (0.4) | 97 (1.8) |
|  | 40-49 | 7250 | 130 (1.8) | 18 (0.2) | 5 (0.1) | 22 (0.3) | 164 (2.3) |
|  | 50-59 | 7961 | 277 (3.5) | 35 (0.4) | 24 (0.3) | 36 (0.5) | 354 (4.4) |
|  | 60-69 | 8366 | 573 (6.8) | 95 (1.1) | 95 (1.1) | 44 (0.5) | 792 (9.5) |
|  | 70-79 | 6288 | 848 (13.5) | 121 (1.9) | 137 (2.2) | 33 (0.5) | 856 (13.6) |
|  | 80+ | 3455 | 726 (21) | 80 (2.3) | 74 (2.1) | 18 (0.5) | 453 (13.1) |
| Blekinge | 1-9 | 15176 | 24 (0.2) | 1 (0) | 2 (0) | 467 (3.1) | 32 (0.2) |
|  | 10-19 | 16381 | 57 (0.3) | 0 (0) | 3 (0) | 297 (1.8) | 124 (0.8) |
|  | 20-29 | 18602 | 88 (0.5) | 6 (0) | 1 (0) | 110 (0.6) | 197 (1.1) |
|  | 30-39 | 16639 | 162 (1) | 18 (0.1) | 2 (0) | 71 (0.4) | 257 (1.5) |
|  | 40-49 | 19941 | 275 (1.4) | 38 (0.2) | 18 (0.1) | 123 (0.6) | 517 (2.6) |
|  | 50-59 | 19340 | 610 (3.2) | 96 (0.5) | 69 (0.4) | 130 (0.7) | 986 (5.1) |
|  | 60-69 | 19965 | 1370 (6.9) | 264 (1.3) | 214 (1.1) | 138 (0.7) | 1957 (9.8) |
|  | 70-79 | 16453 | 2056 (12.5) | 308 (1.9) | 356 (2.2) | 150 (0.9) | 2248 (13.7) |
|  | 80+ | 9639 | 1923 (20) | 221 (2.3) | 230 (2.4) | 117 (1.2) | 1283 (13.3) |
| Skåne | 1-9 | 141556 | 322 (0.2) | 25 (0) | 20 (0) | 4487 (3.2) | 231 (0.2) |
|  | 10-19 | 136630 | 575 (0.4) | 18 (0) | 10 (0) | 2599 (1.9) | 891 (0.7) |
|  | 20-29 | 174415 | 1079 (0.6) | 59 (0) | 22 (0) | 759 (0.4) | 1577 (0.9) |
|  | 30-39 | 164660 | 1430 (0.9) | 165 (0.1) | 34 (0) | 691 (0.4) | 2234 (1.4) |
|  | 40-49 | 171851 | 2860 (1.7) | 404 (0.2) | 140 (0.1) | 995 (0.6) | 4224 (2.5) |
|  | 50-59 | 157164 | 5686 (3.6) | 850 (0.5) | 591 (0.4) | 910 (0.6) | 9030 (5.7) |
|  | 60-69 | 147565 | 11597 (7.9) | 1921 (1.3) | 1673 (1.1) | 1041 (0.7) | 15867 (10.8) |
|  | 70-79 | 112083 | 16185 (14.4) | 2292 (2) | 2721 (2.4) | 975 (0.9) | 16411 (14.6) |
|  | 80+ | 66387 | 14405 (21.7) | 1451 (2.2) | 1784 (2.7) | 615 (0.9) | 8556 (12.9) |
| Halland | 1-9 | 33766 | 83 (0.2) | 2 (0) | 1 (0) | 1506 (4.5) | 52 (0.2) |
|  | 10-19 | 35312 | 145 (0.4) | 5 (0) | 2 (0) | 1097 (3.1) | 259 (0.7) |
|  | 20-29 | 37421 | 185 (0.5) | 10 (0) | 2 (0) | 179 (0.5) | 352 (0.9) |
|  | 30-39 | 34016 | 261 (0.8) | 44 (0.1) | 7 (0) | 148 (0.4) | 423 (1.2) |
|  | 40-49 | 42196 | 558 (1.3) | 108 (0.3) | 24 (0.1) | 215 (0.5) | 849 (2) |
|  | 50-59 | 40052 | 1090 (2.7) | 207 (0.5) | 76 (0.2) | 197 (0.5) | 1763 (4.4) |
|  | 60-69 | 38614 | 2409 (6.2) | 507 (1.3) | 279 (0.7) | 190 (0.5) | 3261 (8.4) |
|  | 70-79 | 29724 | 3747 (12.6) | 582 (2) | 525 (1.8) | 229 (0.8) | 3770 (12.7) |
|  | 80+ | 17475 | 3572 (20.4) | 453 (2.6) | 407 (2.3) | 200 (1.1) | 2131 (12.2) |
| Västra Götaland | 1-9 | 174363 | 432 (0.2) | 28 (0) | 17 (0) | 5613 (3.2) | 312 (0.2) |
|  | 10-19 | 173804 | 613 (0.4) | 16 (0) | 9 (0) | 2649 (1.5) | 1204 (0.7) |
|  | 20-29 | 227114 | 1096 (0.5) | 81 (0) | 14 (0) | 751 (0.3) | 1949 (0.9) |
|  | 30-39 | 205385 | 1608 (0.8) | 218 (0.1) | 31 (0) | 685 (0.3) | 2361 (1.1) |
|  | 40-49 | 216672 | 3288 (1.5) | 492 (0.2) | 118 (0.1) | 869 (0.4) | 5233 (2.4) |
|  | 50-59 | 204787 | 6360 (3.1) | 1023 (0.5) | 500 (0.2) | 988 (0.5) | 10749 (5.2) |
|  | 60-69 | 188492 | 12460 (6.6) | 2247 (1.2) | 1576 (0.8) | 1182 (0.6) | 18743 (9.9) |
|  | 70-79 | 137907 | 17402 (12.6) | 2539 (1.8) | 2613 (1.9) | 1096 (0.8) | 19427 (14.1) |
|  | 80+ | 83660 | 17033 (20.4) | 1784 (2.1) | 1857 (2.2) | 1011 (1.2) | 10767 (12.9) |
| Värmland | 1-9 | 25711 | 74 (0.3) | 1 (0) | 1 (0) | 1017 (4) | 53 (0.2) |
|  | 10-19 | 28202 | 100 (0.4) | 5 (0) | 1 (0) | 565 (2) | 182 (0.6) |
|  | 20-29 | 34737 | 171 (0.5) | 10 (0) | 1 (0) | 174 (0.5) | 345 (1) |
|  | 30-39 | 28276 | 215 (0.8) | 41 (0.1) | 3 (0) | 136 (0.5) | 424 (1.5) |
|  | 40-49 | 34905 | 530 (1.5) | 94 (0.3) | 14 (0) | 215 (0.6) | 1011 (2.9) |
|  | 50-59 | 36072 | 1181 (3.3) | 190 (0.5) | 102 (0.3) | 227 (0.6) | 2134 (5.9) |
|  | 60-69 | 36579 | 2563 (7) | 438 (1.2) | 310 (0.8) | 232 (0.6) | 4271 (11.7) |
|  | 70-79 | 28020 | 3694 (13.2) | 526 (1.9) | 477 (1.7) | 218 (0.8) | 4644 (16.6) |
|  | 80+ | 17626 | 3631 (20.6) | 350 (2) | 334 (1.9) | 153 (0.9) | 2651 (15) |
| Örebro | 1-9 | 30190 | 72 (0.2) | 3 (0) | 3 (0) | 1013 (3.4) | 48 (0.2) |
|  | 10-19 | 31488 | 99 (0.3) | 4 (0) | 1 (0) | 454 (1.4) | 223 (0.7) |
|  | 20-29 | 38926 | 186 (0.5) | 12 (0) | 2 (0) | 97 (0.2) | 368 (0.9) |
|  | 30-39 | 32700 | 244 (0.7) | 36 (0.1) | 5 (0) | 94 (0.3) | 505 (1.5) |
|  | 40-49 | 37379 | 567 (1.5) | 90 (0.2) | 23 (0.1) | 127 (0.3) | 1038 (2.8) |
|  | 50-59 | 35295 | 1104 (3.1) | 142 (0.4) | 97 (0.3) | 158 (0.4) | 2091 (5.9) |
|  | 60-69 | 35638 | 2332 (6.5) | 449 (1.3) | 318 (0.9) | 199 (0.6) | 3686 (10.3) |
|  | 70-79 | 27424 | 3186 (11.6) | 512 (1.9) | 446 (1.6) | 204 (0.7) | 4013 (14.6) |
|  | 80+ | 15534 | 2893 (18.6) | 320 (2.1) | 307 (2) | 161 (1) | 2004 (12.9) |
| Västmanland | 1-9 | 26914 | 55 (0.2) | 3 (0) | 7 (0) | 996 (3.7) | 55 (0.2) |
|  | 10-19 | 28327 | 84 (0.3) | 4 (0) | 2 (0) | 513 (1.8) | 224 (0.8) |
|  | 20-29 | 33453 | 173 (0.5) | 8 (0) | 2 (0) | 88 (0.3) | 332 (1) |
|  | 30-39 | 29465 | 254 (0.9) | 30 (0.1) | 3 (0) | 102 (0.3) | 442 (1.5) |
|  | 40-49 | 34302 | 575 (1.7) | 63 (0.2) | 27 (0.1) | 175 (0.5) | 1016 (3) |
|  | 50-59 | 33375 | 1161 (3.5) | 142 (0.4) | 104 (0.3) | 158 (0.5) | 2030 (6.1) |
|  | 60-69 | 32287 | 2596 (8) | 369 (1.1) | 339 (1) | 228 (0.7) | 3483 (10.8) |
|  | 70-79 | 25477 | 3677 (14.4) | 480 (1.9) | 540 (2.1) | 233 (0.9) | 3771 (14.8) |
|  | 80+ | 14858 | 3304 (22.2) | 329 (2.2) | 314 (2.1) | 199 (1.3) | 2014 (13.6) |
| Dalarna | 1-9 | 27171 | 56 (0.2) | 2 (0) | 2 (0) | 1168 (4.3) | 49 (0.2) |
|  | 10-19 | 29705 | 97 (0.3) | 2 (0) | 0 (0) | 985 (3.3) | 179 (0.6) |
|  | 20-29 | 32842 | 185 (0.6) | 10 (0) | 0 (0) | 132 (0.4) | 345 (1.1) |
|  | 30-39 | 28896 | 269 (0.9) | 28 (0.1) | 2 (0) | 105 (0.4) | 495 (1.7) |
|  | 40-49 | 34436 | 596 (1.7) | 54 (0.2) | 32 (0.1) | 146 (0.4) | 1004 (2.9) |
|  | 50-59 | 36400 | 1392 (3.8) | 141 (0.4) | 83 (0.2) | 175 (0.5) | 2045 (5.6) |
|  | 60-69 | 38984 | 3164 (8.1) | 374 (1) | 297 (0.8) | 221 (0.6) | 4133 (10.6) |
|  | 70-79 | 29040 | 4422 (15.2) | 442 (1.5) | 540 (1.9) | 226 (0.8) | 4443 (15.3) |
|  | 80+ | 17396 | 3880 (22.3) | 313 (1.8) | 347 (2) | 196 (1.1) | 2598 (14.9) |
| Gävleborg | 1-9 | 26806 | 34 (0.1) | 3 (0) | 2 (0) | 1268 (4.7) | 54 (0.2) |
|  | 10-19 | 29791 | 93 (0.3) | 3 (0) | 4 (0) | 742 (2.5) | 222 (0.7) |
|  | 20-29 | 34042 | 227 (0.7) | 12 (0) | 1 (0) | 152 (0.4) | 370 (1.1) |
|  | 30-39 | 28416 | 303 (1.1) | 27 (0.1) | 3 (0) | 133 (0.5) | 394 (1.4) |
|  | 40-49 | 36062 | 663 (1.8) | 86 (0.2) | 22 (0.1) | 126 (0.3) | 1011 (2.8) |
|  | 50-59 | 36573 | 1442 (3.9) | 176 (0.5) | 87 (0.2) | 198 (0.5) | 2143 (5.9) |
|  | 60-69 | 38056 | 3044 (8) | 413 (1.1) | 335 (0.9) | 271 (0.7) | 4152 (10.9) |
|  | 70-79 | 29101 | 4343 (14.9) | 573 (2) | 550 (1.9) | 349 (1.2) | 4331 (14.9) |
|  | 80+ | 16704 | 3896 (23.3) | 324 (1.9) | 409 (2.4) | 269 (1.6) | 2347 (14.1) |
| Västernorrland | 1-9 | 23341 | 50 (0.2) | 2 (0) | 3 (0) | 1032 (4.4) | 56 (0.2) |
|  | 10-19 | 25872 | 91 (0.4) | 0 (0) | 1 (0) | 693 (2.7) | 197 (0.8) |
|  | 20-29 | 28126 | 159 (0.6) | 9 (0) | 5 (0) | 79 (0.3) | 294 (1) |
|  | 30-39 | 25584 | 226 (0.9) | 24 (0.1) | 1 (0) | 81 (0.3) | 397 (1.6) |
|  | 40-49 | 31346 | 463 (1.5) | 60 (0.2) | 9 (0) | 108 (0.3) | 921 (2.9) |
|  | 50-59 | 31343 | 1088 (3.5) | 141 (0.4) | 68 (0.2) | 143 (0.5) | 1939 (6.2) |
|  | 60-69 | 32820 | 2344 (7.1) | 343 (1) | 263 (0.8) | 172 (0.5) | 3834 (11.7) |
|  | 70-79 | 25536 | 3372 (13.2) | 426 (1.7) | 412 (1.6) | 182 (0.7) | 3964 (15.5) |
|  | 80+ | 14804 | 2825 (19.1) | 206 (1.4) | 265 (1.8) | 110 (0.7) | 2179 (14.7) |
| Jämtland | 1-9 | 12444 | 27 (0.2) | 1 (0) | 3 (0) | 337 (2.7) | 20 (0.2) |
|  | 10-19 | 13035 | 41 (0.3) | 1 (0) | 0 (0) | 222 (1.7) | 108 (0.8) |
|  | 20-29 | 15756 | 71 (0.5) | 3 (0) | 3 (0) | 116 (0.7) | 137 (0.9) |
|  | 30-39 | 13427 | 90 (0.7) | 11 (0.1) | 0 (0) | 86 (0.6) | 174 (1.3) |
|  | 40-49 | 15887 | 196 (1.2) | 34 (0.2) | 10 (0.1) | 84 (0.5) | 346 (2.2) |
|  | 50-59 | 16409 | 438 (2.7) | 80 (0.5) | 31 (0.2) | 101 (0.6) | 908 (5.5) |
|  | 60-69 | 17355 | 985 (5.7) | 172 (1) | 125 (0.7) | 120 (0.7) | 1880 (10.8) |
|  | 70-79 | 12653 | 1316 (10.4) | 190 (1.5) | 236 (1.9) | 108 (0.9) | 1968 (15.6) |
|  | 80+ | 7720 | 1226 (15.9) | 103 (1.3) | 168 (2.2) | 77 (1) | 1180 (15.3) |
| Västerbotten | 1-9 | 26267 | 89 (0.3) | 3 (0) | 2 (0) | 1009 (3.8) | 43 (0.2) |
|  | 10-19 | 27265 | 106 (0.4) | 2 (0) | 2 (0) | 476 (1.7) | 219 (0.8) |
|  | 20-29 | 39437 | 169 (0.4) | 12 (0) | 5 (0) | 163 (0.4) | 361 (0.9) |
|  | 30-39 | 30837 | 166 (0.5) | 32 (0.1) | 7 (0) | 130 (0.4) | 423 (1.4) |
|  | 40-49 | 32146 | 427 (1.3) | 56 (0.2) | 17 (0.1) | 165 (0.5) | 784 (2.4) |
|  | 50-59 | 31955 | 990 (3.1) | 143 (0.4) | 58 (0.2) | 216 (0.7) | 1721 (5.4) |
|  | 60-69 | 32344 | 2219 (6.9) | 343 (1.1) | 223 (0.7) | 241 (0.7) | 3154 (9.8) |
|  | 70-79 | 23829 | 3106 (13) | 416 (1.7) | 395 (1.7) | 269 (1.1) | 3285 (13.8) |
|  | 80+ | 14291 | 2906 (20.3) | 219 (1.5) | 248 (1.7) | 191 (1.3) | 1954 (13.7) |
| Norrbotten | 1-9 | 22481 | 42 (0.2) | 0 (0) | 2 (0) | 1188 (5.3) | 44 (0.2) |
|  | 10-19 | 25524 | 79 (0.3) | 2 (0) | 1 (0) | 678 (2.7) | 152 (0.6) |
|  | 20-29 | 32506 | 147 (0.5) | 3 (0) | 5 (0) | 127 (0.4) | 299 (0.9) |
|  | 30-39 | 25488 | 194 (0.8) | 26 (0.1) | 2 (0) | 104 (0.4) | 313 (1.2) |
|  | 40-49 | 31251 | 465 (1.5) | 48 (0.2) | 17 (0.1) | 158 (0.5) | 765 (2.4) |
|  | 50-59 | 33550 | 1231 (3.7) | 123 (0.4) | 84 (0.3) | 244 (0.7) | 1881 (5.6) |
|  | 60-69 | 34194 | 2547 (7.4) | 342 (1) | 314 (0.9) | 288 (0.8) | 3473 (10.2) |
|  | 70-79 | 25619 | 3848 (15) | 382 (1.5) | 539 (2.1) | 306 (1.2) | 3859 (15.1) |
|  | 80+ | 14727 | 3377 (22.9) | 194 (1.3) | 338 (2.3) | 270 (1.8) | 2150 (14.6) |

*. Baseline is on the 1^st^ of January 2016.

**Appendix 6.** Burden and prevalence of underlying medical conditions suggesting high risk of severe COVID-19 in each Swedish county and age group with a five-years look back period.

| County | Age groups  (years) | N | Cardiovascular disease  n (%) | | Cancer  n (%) | COPD  n (%) | Severe asthma  n (%) | Diabetes  n (%) |
| --- | --- | --- | --- | --- | --- | --- | --- | --- |
| Stockholm | 1-9 | 257368 | 1917 (0.7) | 138 (0.1) | | 414 (0.2) | 24401 (9.5) | 389 (0.2) |
|  | 10-19 | 238146 | 2895 (1.2) | 112 (0) | | 265 (0.1) | 12746 (5.4) | 1422 (0.6) |
|  | 20-29 | 301758 | 6425 (2.1) | 405 (0.1) | | 121 (0) | 6388 (2.1) | 2708 (0.9) |
|  | 30-39 | 324475 | 10511 (3.2) | 1083 (0.3) | | 149 (0) | 5859 (1.8) | 4433 (1.4) |
|  | 40-49 | 319742 | 16133 (5) | 2502 (0.8) | | 451 (0.1) | 6450 (2) | 7850 (2.5) |
|  | 50-59 | 271601 | 23886 (8.8) | 5394 (2) | | 1888 (0.7) | 6181 (2.3) | 15250 (5.6) |
|  | 60-69 | 218741 | 36968 (16.9) | 11040 (5) | | 4747 (2.2) | 5598 (2.6) | 22824 (10.4) |
|  | 70-79 | 156684 | 45151 (28.8) | 11912 (7.6) | | 6924 (4.4) | 4679 (3) | 22627 (14.4) |
|  | 80+ | 85524 | 40139 (46.9) | 6912 (8.1) | | 5304 (6.2) | 3008 (3.5) | 12280 (14.4) |
| Uppsala | 1-9 | 37619 | 276 (0.7) | 12 (0) | | 16 (0) | 2965 (7.9) | 76 (0.2) |
|  | 10-19 | 37617 | 414 (1.1) | 16 (0) | | 4 (0) | 1371 (3.6) | 339 (0.9) |
|  | 20-29 | 55691 | 986 (1.8) | 100 (0.2) | | 14 (0) | 1004 (1.8) | 616 (1.1) |
|  | 30-39 | 42875 | 1196 (2.8) | 174 (0.4) | | 16 (0) | 773 (1.8) | 752 (1.8) |
|  | 40-49 | 45498 | 2083 (4.6) | 407 (0.9) | | 62 (0.1) | 866 (1.9) | 1294 (2.8) |
|  | 50-59 | 42280 | 3375 (8) | 815 (1.9) | | 249 (0.6) | 924 (2.2) | 2562 (6.1) |
|  | 60-69 | 40451 | 6235 (15.4) | 1707 (4.2) | | 826 (2) | 962 (2.4) | 4338 (10.7) |
|  | 70-79 | 28724 | 7715 (26.9) | 1962 (6.8) | | 1227 (4.3) | 870 (3) | 4382 (15.3) |
|  | 80+ | 15570 | 6836 (43.9) | 1107 (7.1) | | 764 (4.9) | 659 (4.2) | 2528 (16.2) |
| Södermanland | 1-9 | 29626 | 253 (0.9) | 14 (0) | | 23 (0.1) | 2276 (7.7) | 48 (0.2) |
|  | 10-19 | 31647 | 360 (1.1) | 11 (0) | | 12 (0) | 1324 (4.2) | 214 (0.7) |
|  | 20-29 | 33237 | 682 (2.1) | 26 (0.1) | | 12 (0) | 629 (1.9) | 417 (1.3) |
|  | 30-39 | 30297 | 968 (3.2) | 97 (0.3) | | 22 (0.1) | 448 (1.5) | 558 (1.8) |
|  | 40-49 | 36357 | 1827 (5) | 273 (0.8) | | 86 (0.2) | 557 (1.5) | 1242 (3.4) |
|  | 50-59 | 35708 | 3064 (8.6) | 551 (1.5) | | 332 (0.9) | 740 (2.1) | 2414 (6.8) |
|  | 60-69 | 36434 | 5944 (16.3) | 1403 (3.9) | | 889 (2.4) | 796 (2.2) | 4091 (11.2) |
|  | 70-79 | 28349 | 7707 (27.2) | 1730 (6.1) | | 1334 (4.7) | 820 (2.9) | 4278 (15.1) |
|  | 80+ | 15565 | 6815 (43.8) | 1032 (6.6) | | 885 (5.7) | 581 (3.7) | 2401 (15.4) |
| Östergötland | 1-9 | 46254 | 246 (0.5) | 21 (0) | | 11 (0) | 3609 (7.8) | 87 (0.2) |
|  | 10-19 | 47686 | 433 (0.9) | 27 (0.1) | | 11 (0) | 2151 (4.5) | 300 (0.6) |
|  | 20-29 | 63729 | 1243 (2) | 110 (0.2) | | 23 (0) | 1250 (2) | 760 (1.2) |
|  | 30-39 | 51592 | 1566 (3) | 213 (0.4) | | 18 (0) | 813 (1.6) | 1023 (2) |
|  | 40-49 | 57228 | 2917 (5.1) | 521 (0.9) | | 76 (0.1) | 955 (1.7) | 1736 (3) |
|  | 50-59 | 54206 | 4807 (8.9) | 997 (1.8) | | 391 (0.7) | 1018 (1.9) | 3283 (6.1) |
|  | 60-69 | 52294 | 8989 (17.2) | 2356 (4.5) | | 1152 (2.2) | 1107 (2.1) | 5502 (10.5) |
|  | 70-79 | 39493 | 11411 (28.9) | 3042 (7.7) | | 1676 (4.2) | 1133 (2.9) | 6105 (15.5) |
|  | 80+ | 23992 | 10568 (44) | 1963 (8.2) | | 1129 (4.7) | 851 (3.5) | 3860 (16.1) |
| Jönköping | 1-9 | 37064 | 195 (0.5) | 19 (0.1) | | 44 (0.1) | 3606 (9.7) | 66 (0.2) |
|  | 10-19 | 39202 | 332 (0.8) | 18 (0) | | 9 (0) | 1120 (2.9) | 269 (0.7) |
|  | 20-29 | 44551 | 825 (1.9) | 74 (0.2) | | 16 (0) | 762 (1.7) | 516 (1.2) |
|  | 30-39 | 38982 | 1223 (3.1) | 162 (0.4) | | 19 (0) | 533 (1.4) | 782 (2) |
|  | 40-49 | 44969 | 2477 (5.5) | 376 (0.8) | | 72 (0.2) | 766 (1.7) | 1412 (3.1) |
|  | 50-59 | 42817 | 3816 (8.9) | 828 (1.9) | | 318 (0.7) | 840 (2) | 2670 (6.2) |
|  | 60-69 | 41009 | 6997 (17.1) | 1801 (4.4) | | 936 (2.3) | 964 (2.4) | 4416 (10.8) |
|  | 70-79 | 30933 | 9284 (30) | 2178 (7) | | 1351 (4.4) | 943 (3) | 4989 (16.1) |
|  | 80+ | 19917 | 9429 (47.3) | 1523 (7.6) | | 1005 (5) | 832 (4.2) | 3488 (17.5) |
| Kronoberg | 1-9 | 20056 | 111 (0.6) | 11 (0.1) | | 12 (0.1) | 2422 (12.1) | 33 (0.2) |
|  | 10-19 | 20851 | 225 (1.1) | 7 (0) | | 9 (0) | 1863 (8.9) | 156 (0.7) |
|  | 20-29 | 25096 | 481 (1.9) | 25 (0.1) | | 4 (0) | 570 (2.3) | 325 (1.3) |
|  | 30-39 | 21767 | 622 (2.9) | 70 (0.3) | | 3 (0) | 273 (1.3) | 470 (2.2) |
|  | 40-49 | 24043 | 1193 (5) | 186 (0.8) | | 38 (0.2) | 287 (1.2) | 666 (2.8) |
|  | 50-59 | 22751 | 1884 (8.3) | 434 (1.9) | | 136 (0.6) | 299 (1.3) | 1290 (5.7) |
|  | 60-69 | 23156 | 3785 (16.3) | 1073 (4.6) | | 504 (2.2) | 289 (1.2) | 2325 (10) |
|  | 70-79 | 17275 | 4781 (27.7) | 1260 (7.3) | | 771 (4.5) | 302 (1.7) | 2588 (15) |
|  | 80+ | 11379 | 4925 (43.3) | 900 (7.9) | | 598 (5.3) | 226 (2) | 1776 (15.6) |
| Kalmar | 1-9 | 22045 | 180 (0.8) | 16 (0.1) | | 9 (0) | 2016 (9.1) | 44 (0.2) |
|  | 10-19 | 24031 | 269 (1.1) | 4 (0) | | 1 (0) | 1093 (4.5) | 156 (0.6) |
|  | 20-29 | 28573 | 550 (1.9) | 34 (0.1) | | 10 (0) | 483 (1.7) | 303 (1.1) |
|  | 30-39 | 23800 | 740 (3.1) | 101 (0.4) | | 9 (0) | 291 (1.2) | 394 (1.7) |
|  | 40-49 | 29294 | 1464 (5) | 256 (0.9) | | 58 (0.2) | 410 (1.4) | 760 (2.6) |
|  | 50-59 | 30664 | 2759 (9) | 541 (1.8) | | 246 (0.8) | 494 (1.6) | 1840 (6) |
|  | 60-69 | 32677 | 5624 (17.2) | 1473 (4.5) | | 738 (2.3) | 572 (1.8) | 3565 (10.9) |
|  | 70-79 | 25606 | 7671 (30) | 1770 (6.9) | | 1122 (4.4) | 556 (2.2) | 4218 (16.5) |
|  | 80+ | 15266 | 7018 (46) | 1107 (7.3) | | 791 (5.2) | 383 (2.5) | 2535 (16.6) |
| Gotland | 1-9 | 5251 | 29 (0.6) | 3 (0.1) | | 5 (0.1) | 594 (11.3) | 12 (0.2) |
|  | 10-19 | 5721 | 54 (0.9) | 2 (0) | | 2 (0) | 364 (6.4) | 45 (0.8) |
|  | 20-29 | 6871 | 171 (2.5) | 10 (0.1) | | 0 (0) | 149 (2.2) | 103 (1.5) |
|  | 30-39 | 5476 | 180 (3.3) | 18 (0.3) | | 6 (0.1) | 74 (1.4) | 132 (2.4) |
|  | 40-49 | 7250 | 395 (5.4) | 70 (1) | | 13 (0.2) | 79 (1.1) | 194 (2.7) |
|  | 50-59 | 7961 | 745 (9.4) | 124 (1.6) | | 50 (0.6) | 103 (1.3) | 376 (4.7) |
|  | 60-69 | 8366 | 1443 (17.2) | 325 (3.9) | | 173 (2.1) | 131 (1.6) | 831 (9.9) |
|  | 70-79 | 6288 | 1814 (28.8) | 435 (6.9) | | 256 (4.1) | 114 (1.8) | 909 (14.5) |
|  | 80+ | 3455 | 1542 (44.6) | 274 (7.9) | | 142 (4.1) | 70 (2) | 499 (14.4) |
| Blekinge | 1-9 | 15176 | 81 (0.5) | 10 (0.1) | | 7 (0) | 1245 (8.2) | 58 (0.4) |
|  | 10-19 | 16381 | 168 (1) | 11 (0.1) | | 4 (0) | 796 (4.9) | 157 (1) |
|  | 20-29 | 18602 | 384 (2.1) | 23 (0.1) | | 9 (0) | 394 (2.1) | 291 (1.6) |
|  | 30-39 | 16639 | 575 (3.5) | 67 (0.4) | | 16 (0.1) | 247 (1.5) | 414 (2.5) |
|  | 40-49 | 19941 | 1102 (5.5) | 167 (0.8) | | 51 (0.3) | 323 (1.6) | 652 (3.3) |
|  | 50-59 | 19340 | 1767 (9.1) | 363 (1.9) | | 152 (0.8) | 350 (1.8) | 1152 (6) |
|  | 60-69 | 19965 | 3430 (17.2) | 935 (4.7) | | 438 (2.2) | 417 (2.1) | 2141 (10.7) |
|  | 70-79 | 16453 | 4677 (28.4) | 1192 (7.2) | | 629 (3.8) | 418 (2.5) | 2480 (15.1) |
|  | 80+ | 9639 | 4323 (44.8) | 797 (8.3) | | 442 (4.6) | 306 (3.2) | 1530 (15.9) |
| Skåne | 1-9 | 141556 | 1003 (0.7) | 86 (0.1) | | 105 (0.1) | 12339 (8.7) | 247 (0.2) |
|  | 10-19 | 136630 | 1667 (1.2) | 70 (0.1) | | 35 (0) | 7480 (5.5) | 955 (0.7) |
|  | 20-29 | 174415 | 3700 (2.1) | 229 (0.1) | | 64 (0) | 3466 (2) | 2067 (1.2) |
|  | 30-39 | 164660 | 5599 (3.4) | 610 (0.4) | | 87 (0.1) | 2524 (1.5) | 3551 (2.2) |
|  | 40-49 | 171851 | 9490 (5.5) | 1528 (0.9) | | 351 (0.2) | 2939 (1.7) | 5161 (3) |
|  | 50-59 | 157164 | 15525 (9.9) | 3083 (2) | | 1287 (0.8) | 2757 (1.8) | 10004 (6.4) |
|  | 60-69 | 147565 | 27567 (18.7) | 6811 (4.6) | | 3540 (2.4) | 2942 (2) | 17160 (11.6) |
|  | 70-79 | 112083 | 35121 (31.3) | 8508 (7.6) | | 5368 (4.8) | 2789 (2.5) | 18114 (16.2) |
|  | 80+ | 66387 | 31270 (47.1) | 5276 (7.9) | | 3584 (5.4) | 1870 (2.8) | 10132 (15.3) |
| Halland | 1-9 | 33766 | 239 (0.7) | 16 (0) | | 16 (0) | 2744 (8.1) | 53 (0.2) |
|  | 10-19 | 35312 | 432 (1.2) | 12 (0) | | 5 (0) | 2158 (6.1) | 278 (0.8) |
|  | 20-29 | 37421 | 768 (2.1) | 38 (0.1) | | 4 (0) | 869 (2.3) | 433 (1.2) |
|  | 30-39 | 34016 | 1138 (3.3) | 156 (0.5) | | 15 (0) | 556 (1.6) | 553 (1.6) |
|  | 40-49 | 42196 | 2032 (4.8) | 425 (1) | | 51 (0.1) | 706 (1.7) | 968 (2.3) |
|  | 50-59 | 40052 | 3456 (8.6) | 886 (2.2) | | 198 (0.5) | 680 (1.7) | 1885 (4.7) |
|  | 60-69 | 38614 | 6606 (17.1) | 1968 (5.1) | | 677 (1.8) | 750 (1.9) | 3452 (8.9) |
|  | 70-79 | 29724 | 8864 (29.8) | 2503 (8.4) | | 1092 (3.7) | 790 (2.7) | 4046 (13.6) |
|  | 80+ | 17475 | 8055 (46.1) | 1661 (9.5) | | 784 (4.5) | 629 (3.6) | 2418 (13.8) |
| Västra Götaland | 1-9 | 174363 | 1321 (0.8) | 138 (0.1) | | 92 (0.1) | 12546 (7.2) | 325 (0.2) |
|  | 10-19 | 173804 | 2001 (1.2) | 82 (0) | | 26 (0) | 6625 (3.8) | 1269 (0.7) |
|  | 20-29 | 227114 | 4176 (1.8) | 306 (0.1) | | 72 (0) | 3281 (1.4) | 2241 (1) |
|  | 30-39 | 205385 | 5960 (2.9) | 821 (0.4) | | 98 (0) | 2442 (1.2) | 3052 (1.5) |
|  | 40-49 | 216672 | 10516 (4.9) | 1958 (0.9) | | 322 (0.1) | 2723 (1.3) | 5971 (2.8) |
|  | 50-59 | 204787 | 17314 (8.5) | 3879 (1.9) | | 1262 (0.6) | 2898 (1.4) | 11731 (5.7) |
|  | 60-69 | 188492 | 30332 (16.1) | 8174 (4.3) | | 3437 (1.8) | 3247 (1.7) | 20057 (10.6) |
|  | 70-79 | 137907 | 38804 (28.1) | 9680 (7) | | 5249 (3.8) | 3106 (2.3) | 20962 (15.2) |
|  | 80+ | 83660 | 36850 (44) | 6862 (8.2) | | 3680 (4.4) | 2512 (3) | 12384 (14.8) |
| Värmland | 1-9 | 25711 | 151 (0.6) | 17 (0.1) | | 5 (0) | 2307 (9) | 53 (0.2) |
|  | 10-19 | 28202 | 259 (0.9) | 15 (0.1) | | 1 (0) | 1708 (6.1) | 185 (0.7) |
|  | 20-29 | 34737 | 613 (1.8) | 46 (0.1) | | 8 (0) | 657 (1.9) | 409 (1.2) |
|  | 30-39 | 28276 | 795 (2.8) | 107 (0.4) | | 13 (0) | 479 (1.7) | 579 (2) |
|  | 40-49 | 34905 | 1627 (4.7) | 293 (0.8) | | 31 (0.1) | 547 (1.6) | 1162 (3.3) |
|  | 50-59 | 36072 | 3120 (8.6) | 639 (1.8) | | 212 (0.6) | 662 (1.8) | 2285 (6.3) |
|  | 60-69 | 36579 | 6156 (16.8) | 1528 (4.2) | | 673 (1.8) | 687 (1.9) | 4565 (12.5) |
|  | 70-79 | 28020 | 8212 (29.3) | 1860 (6.6) | | 990 (3.5) | 643 (2.3) | 5015 (17.9) |
|  | 80+ | 17626 | 8009 (45.4) | 1280 (7.3) | | 697 (4) | 466 (2.6) | 3105 (17.6) |
| Örebro | 1-9 | 30190 | 177 (0.6) | 16 (0.1) | | 6 (0) | 2203 (7.3) | 48 (0.2) |
|  | 10-19 | 31488 | 288 (0.9) | 11 (0) | | 7 (0) | 1410 (4.5) | 227 (0.7) |
|  | 20-29 | 38926 | 724 (1.9) | 49 (0.1) | | 8 (0) | 609 (1.6) | 412 (1.1) |
|  | 30-39 | 32700 | 981 (3) | 115 (0.4) | | 10 (0) | 388 (1.2) | 625 (1.9) |
|  | 40-49 | 37379 | 1943 (5.2) | 304 (0.8) | | 61 (0.2) | 549 (1.5) | 1160 (3.1) |
|  | 50-59 | 35295 | 3054 (8.7) | 554 (1.6) | | 216 (0.6) | 552 (1.6) | 2254 (6.4) |
|  | 60-69 | 35638 | 5973 (16.8) | 1342 (3.8) | | 670 (1.9) | 611 (1.7) | 3916 (11) |
|  | 70-79 | 27424 | 7537 (27.5) | 1685 (6.1) | | 872 (3.2) | 608 (2.2) | 4305 (15.7) |
|  | 80+ | 15534 | 6589 (42.4) | 1098 (7.1) | | 609 (3.9) | 458 (2.9) | 2336 (15) |
| Västmanland | 1-9 | 26914 | 246 (0.9) | 22 (0.1) | | 11 (0) | 2102 (7.8) | 57 (0.2) |
|  | 10-19 | 28327 | 299 (1.1) | 8 (0) | | 5 (0) | 1390 (4.9) | 240 (0.8) |
|  | 20-29 | 33453 | 671 (2) | 35 (0.1) | | 5 (0) | 591 (1.8) | 392 (1.2) |
|  | 30-39 | 29465 | 1002 (3.4) | 110 (0.4) | | 12 (0) | 387 (1.3) | 569 (1.9) |
|  | 40-49 | 34302 | 1736 (5.1) | 256 (0.7) | | 62 (0.2) | 522 (1.5) | 1144 (3.3) |
|  | 50-59 | 33375 | 2987 (8.9) | 576 (1.7) | | 208 (0.6) | 535 (1.6) | 2196 (6.6) |
|  | 60-69 | 32287 | 5669 (17.6) | 1342 (4.2) | | 697 (2.2) | 643 (2) | 3722 (11.5) |
|  | 70-79 | 25477 | 7661 (30.1) | 1785 (7) | | 1026 (4) | 666 (2.6) | 4046 (15.9) |
|  | 80+ | 14858 | 6696 (45.1) | 1174 (7.9) | | 622 (4.2) | 545 (3.7) | 2275 (15.3) |
| Dalarna | 1-9 | 27171 | 162 (0.6) | 12 (0) | | 10 (0) | 2503 (9.2) | 51 (0.2) |
|  | 10-19 | 29705 | 259 (0.9) | 14 (0) | | 5 (0) | 2404 (8.1) | 190 (0.6) |
|  | 20-29 | 32842 | 683 (2.1) | 39 (0.1) | | 6 (0) | 800 (2.4) | 428 (1.3) |
|  | 30-39 | 28896 | 945 (3.3) | 101 (0.3) | | 9 (0) | 370 (1.3) | 661 (2.3) |
|  | 40-49 | 34436 | 1757 (5.1) | 280 (0.8) | | 55 (0.2) | 518 (1.5) | 1163 (3.4) |
|  | 50-59 | 36400 | 3404 (9.4) | 586 (1.6) | | 181 (0.5) | 575 (1.6) | 2235 (6.1) |
|  | 60-69 | 38984 | 6859 (17.6) | 1469 (3.8) | | 647 (1.7) | 689 (1.8) | 4376 (11.2) |
|  | 70-79 | 29040 | 8799 (30.3) | 1697 (5.8) | | 1047 (3.6) | 721 (2.5) | 4792 (16.5) |
|  | 80+ | 17396 | 8004 (46) | 1180 (6.8) | | 702 (4) | 552 (3.2) | 2928 (16.8) |
| Gävleborg | 1-9 | 26806 | 132 (0.5) | 14 (0.1) | | 4 (0) | 2429 (9.1) | 57 (0.2) |
|  | 10-19 | 29791 | 243 (0.8) | 14 (0) | | 8 (0) | 1487 (5) | 232 (0.8) |
|  | 20-29 | 34042 | 689 (2) | 39 (0.1) | | 6 (0) | 586 (1.7) | 444 (1.3) |
|  | 30-39 | 28416 | 898 (3.2) | 95 (0.3) | | 7 (0) | 408 (1.4) | 516 (1.8) |
|  | 40-49 | 36062 | 1768 (4.9) | 270 (0.7) | | 45 (0.1) | 448 (1.2) | 1151 (3.2) |
|  | 50-59 | 36573 | 3362 (9.2) | 586 (1.6) | | 205 (0.6) | 578 (1.6) | 2338 (6.4) |
|  | 60-69 | 38056 | 6617 (17.4) | 1410 (3.7) | | 657 (1.7) | 741 (1.9) | 4438 (11.7) |
|  | 70-79 | 29101 | 8656 (29.7) | 1920 (6.6) | | 1048 (3.6) | 866 (3) | 4691 (16.1) |
|  | 80+ | 16704 | 7616 (45.6) | 1180 (7.1) | | 744 (4.5) | 625 (3.7) | 2720 (16.3) |
| Västernorrland | 1-9 | 23341 | 134 (0.6) | 7 (0) | | 10 (0) | 1947 (8.3) | 56 (0.2) |
|  | 10-19 | 25872 | 255 (1) | 10 (0) | | 2 (0) | 1412 (5.5) | 201 (0.8) |
|  | 20-29 | 28126 | 550 (2) | 29 (0.1) | | 10 (0) | 485 (1.7) | 384 (1.4) |
|  | 30-39 | 25584 | 740 (2.9) | 91 (0.4) | | 5 (0) | 323 (1.3) | 543 (2.1) |
|  | 40-49 | 31346 | 1408 (4.5) | 242 (0.8) | | 30 (0.1) | 396 (1.3) | 1057 (3.4) |
|  | 50-59 | 31343 | 2761 (8.8) | 491 (1.6) | | 177 (0.6) | 488 (1.6) | 2058 (6.6) |
|  | 60-69 | 32820 | 5539 (16.9) | 1263 (3.8) | | 529 (1.6) | 540 (1.6) | 4049 (12.3) |
|  | 70-79 | 25536 | 7389 (28.9) | 1570 (6.1) | | 800 (3.1) | 569 (2.2) | 4231 (16.6) |
|  | 80+ | 14804 | 6458 (43.6) | 909 (6.1) | | 527 (3.6) | 400 (2.7) | 2448 (16.5) |
| Jämtland | 1-9 | 12444 | 67 (0.5) | 6 (0) | | 4 (0) | 718 (5.8) | 23 (0.2) |
|  | 10-19 | 13035 | 139 (1.1) | 5 (0) | | 0 (0) | 511 (3.9) | 108 (0.8) |
|  | 20-29 | 15756 | 260 (1.7) | 15 (0.1) | | 6 (0) | 360 (2.3) | 153 (1) |
|  | 30-39 | 13427 | 346 (2.6) | 54 (0.4) | | 4 (0) | 239 (1.8) | 222 (1.7) |
|  | 40-49 | 15887 | 635 (4) | 115 (0.7) | | 23 (0.1) | 254 (1.6) | 404 (2.5) |
|  | 50-59 | 16409 | 1221 (7.4) | 303 (1.8) | | 81 (0.5) | 287 (1.7) | 969 (5.9) |
|  | 60-69 | 17355 | 2548 (14.7) | 715 (4.1) | | 317 (1.8) | 340 (2) | 2014 (11.6) |
|  | 70-79 | 12653 | 3295 (26) | 863 (6.8) | | 475 (3.8) | 341 (2.7) | 2117 (16.7) |
|  | 80+ | 7720 | 3062 (39.7) | 503 (6.5) | | 350 (4.5) | 254 (3.3) | 1340 (17.4) |
| Västerbotten | 1-9 | 26267 | 233 (0.9) | 14 (0.1) | | 23 (0.1) | 2434 (9.3) | 46 (0.2) |
|  | 10-19 | 27265 | 305 (1.1) | 15 (0.1) | | 5 (0) | 1384 (5.1) | 224 (0.8) |
|  | 20-29 | 39437 | 700 (1.8) | 43 (0.1) | | 15 (0) | 852 (2.2) | 415 (1.1) |
|  | 30-39 | 30837 | 740 (2.4) | 99 (0.3) | | 16 (0.1) | 485 (1.6) | 516 (1.7) |
|  | 40-49 | 32146 | 1431 (4.5) | 234 (0.7) | | 43 (0.1) | 591 (1.8) | 881 (2.7) |
|  | 50-59 | 31955 | 2709 (8.5) | 572 (1.8) | | 146 (0.5) | 703 (2.2) | 1875 (5.9) |
|  | 60-69 | 32344 | 5588 (17.3) | 1264 (3.9) | | 494 (1.5) | 762 (2.4) | 3372 (10.4) |
|  | 70-79 | 23829 | 7176 (30.1) | 1547 (6.5) | | 826 (3.5) | 829 (3.5) | 3520 (14.8) |
|  | 80+ | 14291 | 6660 (46.6) | 909 (6.4) | | 561 (3.9) | 636 (4.5) | 2223 (15.6) |
| Norrbotten | 1-9 | 22481 | 93 (0.4) | 7 (0) | | 7 (0) | 2172 (9.7) | 44 (0.2) |
|  | 10-19 | 25524 | 227 (0.9) | 11 (0) | | 3 (0) | 1749 (6.9) | 162 (0.6) |
|  | 20-29 | 32506 | 518 (1.6) | 24 (0.1) | | 16 (0) | 791 (2.4) | 337 (1) |
|  | 30-39 | 25488 | 705 (2.8) | 84 (0.3) | | 9 (0) | 397 (1.6) | 433 (1.7) |
|  | 40-49 | 31251 | 1489 (4.8) | 196 (0.6) | | 43 (0.1) | 653 (2.1) | 891 (2.9) |
|  | 50-59 | 33550 | 3131 (9.3) | 485 (1.4) | | 219 (0.7) | 866 (2.6) | 2038 (6.1) |
|  | 60-69 | 34194 | 6177 (18.1) | 1199 (3.5) | | 712 (2.1) | 1045 (3.1) | 3760 (11) |
|  | 70-79 | 25619 | 8214 (32.1) | 1427 (5.6) | | 1100 (4.3) | 971 (3.8) | 4249 (16.6) |
|  | 80+ | 14727 | 7018 (47.7) | 776 (5.3) | | 703 (4.8) | 716 (4.9) | 2553 (17.3) |

* Baseline is on the 1^st^ of January 2016.

**Appendix 7.** Burden and prevalence of underlying medical conditions suggesting high risk for severe COVID-19 in each Swedish county and age group for a 10-years look back period

| County | Age groups  (years) | Overall,  N | Cardiovascular disease  n (%) | | Cancer  n (%) | COPD  n (%) | Severe asthma  n (%) | Diabetes  n (%) |
| --- | --- | --- | --- | --- | --- | --- | --- | --- |
| Stockholm | 1-9 | 257368 | 2308 (0.9) | 198 (0.1) | | 458 (0.2) | 29979 (11.6) | 403 (0.2) |
|  | 10-19 | 238146 | 4362 (1.8) | 210 (0.1) | | 343 (0.1) | 21814 (9.2) | 1479 (0.6) |
|  | 20-29 | 301758 | 9537 (3.2) | 581 (0.2) | | 195 (0.1) | 11992 (4) | 2890 (1) |
|  | 30-39 | 324475 | 16170 (5) | 1647 (0.5) | | 223 (0.1) | 8473 (2.6) | 5027 (1.5) |
|  | 40-49 | 319742 | 25003 (7.8) | 3821 (1.2) | | 637 (0.2) | 9214 (2.9) | 8746 (2.7) |
|  | 50-59 | 271601 | 34707 (12.8) | 8060 (3) | | 2405 (0.9) | 8686 (3.2) | 15988 (5.9) |
|  | 60-69 | 218741 | 49422 (22.6) | 16440 (7.5) | | 5804 (2.7) | 7682 (3.5) | 23848 (10.9) |
|  | 70-79 | 156684 | 57185 (36.5) | 19257 (12.3) | | 8226 (5.3) | 6405 (4.1) | 23761 (15.2) |
|  | 80+ | 85524 | 47884 (56) | 11463 (13.4) | | 6220 (7.3) | 4093 (4.8) | 13315 (15.6) |
| Uppsala | 1-9 | 37619 | 334 (0.9) | 20 (0.1) | | 19 (0.1) | 3469 (9.2) | 78 (0.2) |
|  | 10-19 | 37617 | 590 (1.6) | 30 (0.1) | | 8 (0) | 2180 (5.8) | 342 (0.9) |
|  | 20-29 | 55691 | 1538 (2.8) | 136 (0.2) | | 19 (0) | 1816 (3.3) | 638 (1.1) |
|  | 30-39 | 42875 | 1863 (4.3) | 232 (0.5) | | 28 (0.1) | 1159 (2.7) | 867 (2) |
|  | 40-49 | 45498 | 3142 (6.9) | 586 (1.3) | | 82 (0.2) | 1308 (2.9) | 1471 (3.2) |
|  | 50-59 | 42280 | 4744 (11.2) | 1180 (2.8) | | 324 (0.8) | 1315 (3.1) | 2686 (6.4) |
|  | 60-69 | 40451 | 8348 (20.6) | 2623 (6.5) | | 997 (2.5) | 1351 (3.3) | 4479 (11.1) |
|  | 70-79 | 28724 | 9918 (34.5) | 3125 (10.9) | | 1434 (5) | 1134 (3.9) | 4534 (15.8) |
|  | 80+ | 15570 | 8376 (53.8) | 1809 (11.6) | | 871 (5.6) | 855 (5.5) | 2638 (16.9) |
| Södermanland | 1-9 | 29626 | 308 (1) | 17 (0.1) | | 27 (0.1) | 2843 (9.6) | 50 (0.2) |
|  | 10-19 | 31647 | 512 (1.6) | 23 (0.1) | | 20 (0.1) | 2403 (7.6) | 219 (0.7) |
|  | 20-29 | 33237 | 1009 (3) | 37 (0.1) | | 16 (0) | 1232 (3.7) | 438 (1.3) |
|  | 30-39 | 30297 | 1456 (4.8) | 143 (0.5) | | 31 (0.1) | 699 (2.3) | 636 (2.1) |
|  | 40-49 | 36357 | 2785 (7.7) | 409 (1.1) | | 111 (0.3) | 780 (2.1) | 1341 (3.7) |
|  | 50-59 | 35708 | 4379 (12.3) | 847 (2.4) | | 400 (1.1) | 991 (2.8) | 2479 (6.9) |
|  | 60-69 | 36434 | 7898 (21.7) | 2153 (5.9) | | 1051 (2.9) | 1072 (2.9) | 4163 (11.4) |
|  | 70-79 | 28349 | 9727 (34.3) | 2946 (10.4) | | 1524 (5.4) | 1048 (3.7) | 4399 (15.5) |
|  | 80+ | 15565 | 8191 (52.6) | 1747 (11.2) | | 1001 (6.4) | 721 (4.6) | 2505 (16.1) |
| Östergötland | 1-9 | 46254 | 308 (0.7) | 34 (0.1) | | 16 (0) | 4484 (9.7) | 89 (0.2) |
|  | 10-19 | 47686 | 647 (1.4) | 54 (0.1) | | 22 (0) | 3689 (7.7) | 309 (0.6) |
|  | 20-29 | 63729 | 1949 (3.1) | 154 (0.2) | | 33 (0.1) | 2546 (4) | 783 (1.2) |
|  | 30-39 | 51592 | 2381 (4.6) | 302 (0.6) | | 25 (0) | 1149 (2.2) | 1204 (2.3) |
|  | 40-49 | 57228 | 4371 (7.6) | 771 (1.3) | | 99 (0.2) | 1331 (2.3) | 1949 (3.4) |
|  | 50-59 | 54206 | 6804 (12.6) | 1545 (2.9) | | 478 (0.9) | 1372 (2.5) | 3379 (6.2) |
|  | 60-69 | 52294 | 11848 (22.7) | 3479 (6.7) | | 1383 (2.6) | 1510 (2.9) | 5654 (10.8) |
|  | 70-79 | 39493 | 14540 (36.8) | 4750 (12) | | 1932 (4.9) | 1493 (3.8) | 6303 (16) |
|  | 80+ | 23992 | 12910 (53.8) | 3139 (13.1) | | 1311 (5.5) | 1078 (4.5) | 4054 (16.9) |
| Jönköping | 1-9 | 37064 | 243 (0.7) | 31 (0.1) | | 49 (0.1) | 3901 (10.5) | 67 (0.2) |
|  | 10-19 | 39202 | 482 (1.2) | 31 (0.1) | | 12 (0) | 1824 (4.7) | 272 (0.7) |
|  | 20-29 | 44551 | 1201 (2.7) | 103 (0.2) | | 23 (0.1) | 1301 (2.9) | 537 (1.2) |
|  | 30-39 | 38982 | 1891 (4.9) | 234 (0.6) | | 27 (0.1) | 775 (2) | 980 (2.5) |
|  | 40-49 | 44969 | 3725 (8.3) | 572 (1.3) | | 91 (0.2) | 1049 (2.3) | 1607 (3.6) |
|  | 50-59 | 42817 | 5519 (12.9) | 1222 (2.9) | | 388 (0.9) | 1134 (2.6) | 2732 (6.4) |
|  | 60-69 | 41009 | 9415 (23) | 2732 (6.7) | | 1104 (2.7) | 1286 (3.1) | 4526 (11) |
|  | 70-79 | 30933 | 11630 (37.6) | 3646 (11.8) | | 1539 (5) | 1233 (4) | 5103 (16.5) |
|  | 80+ | 19917 | 11187 (56.2) | 2510 (12.6) | | 1136 (5.7) | 1043 (5.2) | 3617 (18.2) |
| Kronoberg | 1-9 | 20056 | 149 (0.7) | 12 (0.1) | | 13 (0.1) | 2684 (13.4) | 33 (0.2) |
|  | 10-19 | 20851 | 377 (1.8) | 18 (0.1) | | 11 (0.1) | 2753 (13.2) | 157 (0.8) |
|  | 20-29 | 25096 | 750 (3) | 41 (0.2) | | 6 (0) | 1326 (5.3) | 337 (1.3) |
|  | 30-39 | 21767 | 956 (4.4) | 105 (0.5) | | 10 (0) | 420 (1.9) | 554 (2.5) |
|  | 40-49 | 24043 | 1803 (7.5) | 271 (1.1) | | 61 (0.3) | 414 (1.7) | 760 (3.2) |
|  | 50-59 | 22751 | 2819 (12.4) | 653 (2.9) | | 170 (0.7) | 436 (1.9) | 1334 (5.9) |
|  | 60-69 | 23156 | 5089 (22) | 1637 (7.1) | | 591 (2.6) | 416 (1.8) | 2391 (10.3) |
|  | 70-79 | 17275 | 6179 (35.8) | 2136 (12.4) | | 880 (5.1) | 417 (2.4) | 2660 (15.4) |
|  | 80+ | 11379 | 6036 (53) | 1527 (13.4) | | 691 (6.1) | 307 (2.7) | 1868 (16.4) |
| Kalmar | 1-9 | 22045 | 225 (1) | 20 (0.1) | | 12 (0.1) | 2279 (10.3) | 44 (0.2) |
|  | 10-19 | 24031 | 438 (1.8) | 9 (0) | | 4 (0) | 1798 (7.5) | 157 (0.7) |
|  | 20-29 | 28573 | 843 (3) | 47 (0.2) | | 17 (0.1) | 951 (3.3) | 326 (1.1) |
|  | 30-39 | 23800 | 1159 (4.9) | 133 (0.6) | | 13 (0.1) | 446 (1.9) | 501 (2.1) |
|  | 40-49 | 29294 | 2351 (8) | 378 (1.3) | | 77 (0.3) | 588 (2) | 873 (3) |
|  | 50-59 | 30664 | 4081 (13.3) | 872 (2.8) | | 298 (1) | 678 (2.2) | 1902 (6.2) |
|  | 60-69 | 32677 | 7673 (23.5) | 2291 (7) | | 871 (2.7) | 744 (2.3) | 3637 (11.1) |
|  | 70-79 | 25606 | 9774 (38.2) | 2894 (11.3) | | 1294 (5.1) | 715 (2.8) | 4323 (16.9) |
|  | 80+ | 15266 | 8419 (55.1) | 1882 (12.3) | | 891 (5.8) | 476 (3.1) | 2642 (17.3) |
| Gotland | 1-9 | 5251 | 35 (0.7) | 6 (0.1) | | 5 (0.1) | 744 (14.2) | 12 (0.2) |
|  | 10-19 | 5721 | 86 (1.5) | 4 (0.1) | | 3 (0.1) | 575 (10.1) | 46 (0.8) |
|  | 20-29 | 6871 | 234 (3.4) | 14 (0.2) | | 1 (0) | 311 (4.5) | 110 (1.6) |
|  | 30-39 | 5476 | 285 (5.2) | 31 (0.6) | | 6 (0.1) | 112 (2) | 152 (2.8) |
|  | 40-49 | 7250 | 598 (8.2) | 106 (1.5) | | 19 (0.3) | 138 (1.9) | 225 (3.1) |
|  | 50-59 | 7961 | 1049 (13.2) | 184 (2.3) | | 63 (0.8) | 148 (1.9) | 394 (4.9) |
|  | 60-69 | 8366 | 1962 (23.5) | 504 (6) | | 209 (2.5) | 192 (2.3) | 857 (10.2) |
|  | 70-79 | 6288 | 2338 (37.2) | 693 (11) | | 302 (4.8) | 150 (2.4) | 933 (14.8) |
|  | 80+ | 3455 | 1858 (53.8) | 430 (12.4) | | 176 (5.1) | 105 (3) | 517 (15) |
| Blekinge | 1-9 | 15176 | 108 (0.7) | 16 (0.1) | | 7 (0) | 1550 (10.2) | 60 (0.4) |
|  | 10-19 | 16381 | 233 (1.4) | 19 (0.1) | | 5 (0) | 1415 (8.6) | 161 (1) |
|  | 20-29 | 18602 | 588 (3.2) | 36 (0.2) | | 19 (0.1) | 765 (4.1) | 300 (1.6) |
|  | 30-39 | 16639 | 910 (5.5) | 92 (0.6) | | 22 (0.1) | 368 (2.2) | 482 (2.9) |
|  | 40-49 | 19941 | 1707 (8.6) | 265 (1.3) | | 71 (0.4) | 463 (2.3) | 729 (3.7) |
|  | 50-59 | 19340 | 2647 (13.7) | 548 (2.8) | | 198 (1) | 477 (2.5) | 1190 (6.2) |
|  | 60-69 | 19965 | 4743 (23.8) | 1393 (7) | | 536 (2.7) | 538 (2.7) | 2191 (11) |
|  | 70-79 | 16453 | 6084 (37) | 1952 (11.9) | | 738 (4.5) | 521 (3.2) | 2540 (15.4) |
|  | 80+ | 9639 | 5232 (54.3) | 1274 (13.2) | | 509 (5.3) | 362 (3.8) | 1576 (16.4) |
| Skåne | 1-9 | 141556 | 1292 (0.9) | 116 (0.1) | | 130 (0.1) | 14875 (10.5) | 250 (0.2) |
|  | 10-19 | 136630 | 2495 (1.8) | 120 (0.1) | | 57 (0) | 12657 (9.3) | 972 (0.7) |
|  | 20-29 | 174415 | 5700 (3.3) | 322 (0.2) | | 102 (0.1) | 7405 (4.2) | 2229 (1.3) |
|  | 30-39 | 164660 | 8466 (5.1) | 910 (0.6) | | 146 (0.1) | 4061 (2.5) | 4309 (2.6) |
|  | 40-49 | 171851 | 14214 (8.3) | 2202 (1.3) | | 487 (0.3) | 4469 (2.6) | 6115 (3.6) |
|  | 50-59 | 157164 | 21693 (13.8) | 4705 (3) | | 1731 (1.1) | 4093 (2.6) | 10457 (6.7) |
|  | 60-69 | 147565 | 36155 (24.5) | 10407 (7.1) | | 4417 (3) | 4218 (2.9) | 17714 (12) |
|  | 70-79 | 112083 | 43676 (39) | 13628 (12.2) | | 6433 (5.7) | 3949 (3.5) | 18775 (16.8) |
|  | 80+ | 66387 | 37517 (56.5) | 8795 (13.2) | | 4289 (6.5) | 2570 (3.9) | 10683 (16.1) |
| Halland | 1-9 | 33766 | 300 (0.9) | 25 (0.1) | | 22 (0.1) | 3193 (9.5) | 54 (0.2) |
|  | 10-19 | 35312 | 691 (2) | 27 (0.1) | | 13 (0) | 3401 (9.6) | 284 (0.8) |
|  | 20-29 | 37421 | 1239 (3.3) | 64 (0.2) | | 8 (0) | 1850 (4.9) | 458 (1.2) |
|  | 30-39 | 34016 | 1735 (5.1) | 225 (0.7) | | 22 (0.1) | 900 (2.6) | 662 (1.9) |
|  | 40-49 | 42196 | 3241 (7.7) | 580 (1.4) | | 70 (0.2) | 1121 (2.7) | 1127 (2.7) |
|  | 50-59 | 40052 | 5147 (12.9) | 1298 (3.2) | | 276 (0.7) | 1079 (2.7) | 1961 (4.9) |
|  | 60-69 | 38614 | 8974 (23.2) | 2930 (7.6) | | 852 (2.2) | 1108 (2.9) | 3539 (9.2) |
|  | 70-79 | 29724 | 11402 (38.4) | 3913 (13.2) | | 1350 (4.5) | 1149 (3.9) | 4158 (14) |
|  | 80+ | 17475 | 9820 (56.2) | 2690 (15.4) | | 958 (5.5) | 871 (5) | 2532 (14.5) |
| Västra Götaland | 1-9 | 174363 | 1630 (0.9) | 187 (0.1) | | 114 (0.1) | 16001 (9.2) | 334 (0.2) |
|  | 10-19 | 173804 | 3271 (1.9) | 152 (0.1) | | 51 (0) | 11038 (6.4) | 1299 (0.7) |
|  | 20-29 | 227114 | 6526 (2.9) | 434 (0.2) | | 112 (0) | 6827 (3) | 2379 (1) |
|  | 30-39 | 205385 | 9127 (4.4) | 1199 (0.6) | | 154 (0.1) | 3939 (1.9) | 3552 (1.7) |
|  | 40-49 | 216672 | 16236 (7.5) | 2895 (1.3) | | 449 (0.2) | 4194 (1.9) | 6701 (3.1) |
|  | 50-59 | 204787 | 25296 (12.4) | 5856 (2.9) | | 1672 (0.8) | 4317 (2.1) | 12193 (6) |
|  | 60-69 | 188492 | 41278 (21.9) | 12611 (6.7) | | 4200 (2.2) | 4613 (2.4) | 20575 (10.9) |
|  | 70-79 | 137907 | 49939 (36.2) | 15968 (11.6) | | 6327 (4.6) | 4403 (3.2) | 21532 (15.6) |
|  | 80+ | 83660 | 45020 (53.8) | 11177 (13.4) | | 4421 (5.3) | 3368 (4) | 13016 (15.6) |
| Värmland | 1-9 | 25711 | 181 (0.7) | 23 (0.1) | | 12 (0) | 2557 (9.9) | 55 (0.2) |
|  | 10-19 | 28202 | 373 (1.3) | 29 (0.1) | | 2 (0) | 2358 (8.4) | 188 (0.7) |
|  | 20-29 | 34737 | 868 (2.5) | 58 (0.2) | | 14 (0) | 1156 (3.3) | 428 (1.2) |
|  | 30-39 | 28276 | 1170 (4.1) | 160 (0.6) | | 16 (0.1) | 604 (2.1) | 668 (2.4) |
|  | 40-49 | 34905 | 2394 (6.9) | 428 (1.2) | | 39 (0.1) | 700 (2) | 1286 (3.7) |
|  | 50-59 | 36072 | 4312 (12) | 958 (2.7) | | 252 (0.7) | 832 (2.3) | 2352 (6.5) |
|  | 60-69 | 36579 | 7992 (21.8) | 2256 (6.2) | | 774 (2.1) | 861 (2.4) | 4654 (12.7) |
|  | 70-79 | 28020 | 10192 (36.4) | 2992 (10.7) | | 1128 (4) | 831 (3) | 5155 (18.4) |
|  | 80+ | 17626 | 9431 (53.5) | 2061 (11.7) | | 791 (4.5) | 585 (3.3) | 3239 (18.4) |
| Örebro | 1-9 | 30190 | 216 (0.7) | 23 (0.1) | | 8 (0) | 2497 (8.3) | 49 (0.2) |
|  | 10-19 | 31488 | 411 (1.3) | 20 (0.1) | | 10 (0) | 2281 (7.2) | 228 (0.7) |
|  | 20-29 | 38926 | 1108 (2.8) | 70 (0.2) | | 14 (0) | 1461 (3.8) | 427 (1.1) |
|  | 30-39 | 32700 | 1409 (4.3) | 174 (0.5) | | 15 (0) | 589 (1.8) | 684 (2.1) |
|  | 40-49 | 37379 | 2727 (7.3) | 441 (1.2) | | 77 (0.2) | 752 (2) | 1232 (3.3) |
|  | 50-59 | 35295 | 4256 (12.1) | 877 (2.5) | | 258 (0.7) | 788 (2.2) | 2318 (6.6) |
|  | 60-69 | 35638 | 7927 (22.2) | 2089 (5.9) | | 851 (2.4) | 906 (2.5) | 3994 (11.2) |
|  | 70-79 | 27424 | 9677 (35.3) | 2826 (10.3) | | 1063 (3.9) | 849 (3.1) | 4403 (16.1) |
|  | 80+ | 15534 | 8125 (52.3) | 1815 (11.7) | | 721 (4.6) | 591 (3.8) | 2445 (15.7) |
| Västmanland | 1-9 | 26914 | 283 (1.1) | 30 (0.1) | | 20 (0.1) | 2435 (9) | 59 (0.2) |
|  | 10-19 | 28327 | 413 (1.5) | 16 (0.1) | | 9 (0) | 2333 (8.2) | 244 (0.9) |
|  | 20-29 | 33453 | 1046 (3.1) | 60 (0.2) | | 12 (0) | 1291 (3.9) | 409 (1.2) |
|  | 30-39 | 29465 | 1533 (5.2) | 175 (0.6) | | 23 (0.1) | 629 (2.1) | 619 (2.1) |
|  | 40-49 | 34302 | 2730 (8) | 393 (1.1) | | 89 (0.3) | 790 (2.3) | 1226 (3.6) |
|  | 50-59 | 33375 | 4415 (13.2) | 880 (2.6) | | 287 (0.9) | 790 (2.4) | 2266 (6.8) |
|  | 60-69 | 32287 | 7694 (23.8) | 2080 (6.4) | | 831 (2.6) | 889 (2.8) | 3809 (11.8) |
|  | 70-79 | 25477 | 9734 (38.2) | 2881 (11.3) | | 1191 (4.7) | 892 (3.5) | 4127 (16.2) |
|  | 80+ | 14858 | 8142 (54.8) | 1856 (12.5) | | 737 (5) | 689 (4.6) | 2354 (15.8) |
| Dalarna | 1-9 | 27171 | 209 (0.8) | 16 (0.1) | | 19 (0.1) | 3010 (11.1) | 51 (0.2) |
|  | 10-19 | 29705 | 399 (1.3) | 24 (0.1) | | 10 (0) | 3905 (13.1) | 193 (0.6) |
|  | 20-29 | 32842 | 1059 (3.2) | 52 (0.2) | | 15 (0) | 2024 (6.2) | 443 (1.3) |
|  | 30-39 | 28896 | 1457 (5) | 144 (0.5) | | 15 (0.1) | 670 (2.3) | 736 (2.5) |
|  | 40-49 | 34436 | 2806 (8.1) | 411 (1.2) | | 80 (0.2) | 801 (2.3) | 1258 (3.7) |
|  | 50-59 | 36400 | 4995 (13.7) | 894 (2.5) | | 222 (0.6) | 877 (2.4) | 2290 (6.3) |
|  | 60-69 | 38984 | 9377 (24.1) | 2222 (5.7) | | 808 (2.1) | 992 (2.5) | 4478 (11.5) |
|  | 70-79 | 29040 | 11204 (38.6) | 2879 (9.9) | | 1249 (4.3) | 956 (3.3) | 4929 (17) |
|  | 80+ | 17396 | 9716 (55.9) | 1946 (11.2) | | 820 (4.7) | 703 (4) | 3056 (17.6) |
| Gävleborg | 1-9 | 26806 | 175 (0.7) | 19 (0.1) | | 11 (0) | 2918 (10.9) | 58 (0.2) |
|  | 10-19 | 29791 | 395 (1.3) | 19 (0.1) | | 11 (0) | 2607 (8.8) | 235 (0.8) |
|  | 20-29 | 34042 | 1027 (3) | 55 (0.2) | | 9 (0) | 1226 (3.6) | 459 (1.3) |
|  | 30-39 | 28416 | 1357 (4.8) | 143 (0.5) | | 11 (0) | 561 (2) | 588 (2.1) |
|  | 40-49 | 36062 | 2702 (7.5) | 396 (1.1) | | 59 (0.2) | 666 (1.8) | 1284 (3.6) |
|  | 50-59 | 36573 | 4826 (13.2) | 882 (2.4) | | 248 (0.7) | 785 (2.1) | 2428 (6.6) |
|  | 60-69 | 38056 | 8881 (23.3) | 2127 (5.6) | | 758 (2) | 997 (2.6) | 4532 (11.9) |
|  | 70-79 | 29101 | 11010 (37.8) | 2975 (10.2) | | 1190 (4.1) | 1070 (3.7) | 4813 (16.5) |
|  | 80+ | 16704 | 9123 (54.6) | 1883 (11.3) | | 844 (5.1) | 741 (4.4) | 2837 (17) |
| Västernorrland | 1-9 | 23341 | 183 (0.8) | 11 (0) | | 21 (0.1) | 2299 (9.8) | 57 (0.2) |
|  | 10-19 | 25872 | 367 (1.4) | 23 (0.1) | | 6 (0) | 2437 (9.4) | 206 (0.8) |
|  | 20-29 | 28126 | 821 (2.9) | 38 (0.1) | | 16 (0.1) | 1148 (4.1) | 409 (1.5) |
|  | 30-39 | 25584 | 1108 (4.3) | 137 (0.5) | | 8 (0) | 512 (2) | 663 (2.6) |
|  | 40-49 | 31346 | 2134 (6.8) | 353 (1.1) | | 44 (0.1) | 603 (1.9) | 1215 (3.9) |
|  | 50-59 | 31343 | 3891 (12.4) | 729 (2.3) | | 221 (0.7) | 677 (2.2) | 2128 (6.8) |
|  | 60-69 | 32820 | 7513 (22.9) | 1909 (5.8) | | 636 (1.9) | 755 (2.3) | 4118 (12.5) |
|  | 70-79 | 25536 | 9350 (36.6) | 2552 (10) | | 930 (3.6) | 788 (3.1) | 4316 (16.9) |
|  | 80+ | 14804 | 7858 (53.1) | 1564 (10.6) | | 611 (4.1) | 537 (3.6) | 2543 (17.2) |
| Jämtland | 1-9 | 12444 | 90 (0.7) | 10 (0.1) | | 10 (0.1) | 859 (6.9) | 23 (0.2) |
|  | 10-19 | 13035 | 206 (1.6) | 6 (0) | | 3 (0) | 935 (7.2) | 111 (0.9) |
|  | 20-29 | 15756 | 419 (2.7) | 21 (0.1) | | 8 (0.1) | 651 (4.1) | 159 (1) |
|  | 30-39 | 13427 | 528 (3.9) | 76 (0.6) | | 6 (0) | 327 (2.4) | 250 (1.9) |
|  | 40-49 | 15887 | 1005 (6.3) | 163 (1) | | 32 (0.2) | 356 (2.2) | 433 (2.7) |
|  | 50-59 | 16409 | 1779 (10.8) | 454 (2.8) | | 113 (0.7) | 398 (2.4) | 992 (6) |
|  | 60-69 | 17355 | 3444 (19.8) | 1092 (6.3) | | 386 (2.2) | 457 (2.6) | 2047 (11.8) |
|  | 70-79 | 12653 | 4157 (32.9) | 1382 (10.9) | | 560 (4.4) | 440 (3.5) | 2157 (17) |
|  | 80+ | 7720 | 3731 (48.3) | 854 (11.1) | | 403 (5.2) | 324 (4.2) | 1376 (17.8) |
| Västerbotten | 1-9 | 26267 | 290 (1.1) | 18 (0.1) | | 25 (0.1) | 2934 (11.2) | 46 (0.2) |
|  | 10-19 | 27265 | 471 (1.7) | 26 (0.1) | | 10 (0) | 2698 (9.9) | 227 (0.8) |
|  | 20-29 | 39437 | 1102 (2.8) | 71 (0.2) | | 21 (0.1) | 1931 (4.9) | 431 (1.1) |
|  | 30-39 | 30837 | 1287 (4.2) | 150 (0.5) | | 21 (0.1) | 810 (2.6) | 593 (1.9) |
|  | 40-49 | 32146 | 2230 (6.9) | 365 (1.1) | | 64 (0.2) | 941 (2.9) | 963 (3) |
|  | 50-59 | 31955 | 3922 (12.3) | 890 (2.8) | | 199 (0.6) | 1089 (3.4) | 1918 (6) |
|  | 60-69 | 32344 | 7500 (23.2) | 1936 (6) | | 634 (2) | 1161 (3.6) | 3446 (10.7) |
|  | 70-79 | 23829 | 9166 (38.5) | 2508 (10.5) | | 1029 (4.3) | 1233 (5.2) | 3615 (15.2) |
|  | 80+ | 14291 | 8164 (57.1) | 1630 (11.4) | | 687 (4.8) | 896 (6.3) | 2324 (16.3) |
| Norrbotten | 1-9 | 22481 | 123 (0.5) | 9 (0) | | 12 (0.1) | 2471 (11) | 47 (0.2) |
|  | 10-19 | 25524 | 335 (1.3) | 23 (0.1) | | 6 (0) | 2676 (10.5) | 176 (0.7) |
|  | 20-29 | 32506 | 877 (2.7) | 41 (0.1) | | 26 (0.1) | 1800 (5.5) | 373 (1.1) |
|  | 30-39 | 25488 | 1130 (4.4) | 130 (0.5) | | 21 (0.1) | 637 (2.5) | 544 (2.1) |
|  | 40-49 | 31251 | 2366 (7.6) | 292 (0.9) | | 74 (0.2) | 959 (3.1) | 1065 (3.4) |
|  | 50-59 | 33550 | 4624 (13.8) | 739 (2.2) | | 299 (0.9) | 1244 (3.7) | 2180 (6.5) |
|  | 60-69 | 34194 | 8482 (24.8) | 1788 (5.2) | | 932 (2.7) | 1481 (4.3) | 3968 (11.6) |
|  | 70-79 | 25619 | 10471 (40.9) | 2298 (9) | | 1337 (5.2) | 1329 (5.2) | 4454 (17.4) |
|  | 80+ | 14727 | 8499 (57.7) | 1364 (9.3) | | 850 (5.8) | 1. .5) | 1. .7) |

* Baseline is on the 1^st^ of January 2016.

**Appendix 8.** Burden and prevalence of at least one, two, and three prognostic factors for severe COVID-19 in each Swedish county for a one-year look back period

| County | Prognostic factors, n (%) | | |
| --- | --- | --- | --- |
|  | **At least one** | **At least two** | **At least three** |
| Stockholm | 352718 (16.2) | 78994 (3.6) | 14796 (0.7) |
| Uppsala | 62420 (18) | 14013 (4) | 2707 (0.8) |
| Södermanland | 58797 (21.2) | 13198 (4.8) | 2380 (0.9) |
| Östergötland | 86974 (19.9) | 20396 (4.7) | 3907 (0.9) |
| Jönköping | 69633 (20.5) | 17470 (5.1) | 3678 (1.1) |
| Kronoberg | 38543 (20.7) | 8603 (4.6) | 1608 (0.9) |
| Kalmar | 53529 (23.1) | 12964 (5.6) | 2600 (1.1) |
| Gotland | 13023 (23) | 3050 (5.4) | 511 (0.9) |
| Blekinge | 34016 (22.4) | 7966 (5.2) | 1517 (1) |
| Skåne | 246832 (19.4) | 59511 (4.7) | 11103 (0.9) |
| Halland | 62532 (20.3) | 13978 (4.5) | 2497 (0.8) |
| Västra Götaland | 300300 (18.6) | 68489 (4.2) | 12306 (0.8) |
| Värmland | 61313 (22.7) | 15053 (5.6) | 2684 (1) |
| Örebro | 57565 (20.2) | 12871 (4.5) | 2213 (0.8) |
| Västmanland | 54737 (21.2) | 13431 (5.2) | 2560 (1) |
| Dalarna | 62866 (22.9) | 15699 (5.7) | 2937 (1.1) |
| Gävleborg | 62402 (22.6) | 15644 (5.7) | 2978 (1.1) |
| Västernorrland | 54376 (22.8) | 12789 (5.4) | 2168 (0.9) |
| Jämtland | 26788 (21.5) | 5990 (4.8) | 1017 (0.8) |
| Västerbotten | 51198 (19.8) | 11786 (4.6) | 2083 (0.8) |
| Norrbotten | 54305 (22.1) | 13597 (5.5) | 2743 (1.1) |

*On the 1^st^ of January 2016

**Appendix 9.** Burden and prevalence of at least one, two, and three prognostic factors for severe COVID-19 in each Swedish county for a five-years look back period*

| County | Prognostic factors, n (%) | | |
| --- | --- | --- | --- |
|  | **At least one** | **At least two** | **At least three** |
| Stockholm | 464589 (21.4) | 142815 (6.6) | 36899 (1.7) |
| Uppsala | 77647 (22.4) | 24789 (7.2) | 6553 (1.9) |
| Södermanland | 72257 (26.1) | 24208 (8.7) | 6441 (2.3) |
| Östergötland | 107010 (24.5) | 36333 (8.3) | 9711 (2.2) |
| Jönköping | 85183 (25.1) | 30029 (8.8) | 8354 (2.5) |
| Kronoberg | 48445 (26) | 15686 (8.4) | 4041 (2.2) |
| Kalmar | 64669 (27.9) | 23447 (10.1) | 6352 (2.7) |
| Gotland | 15941 (28.1) | 5364 (9.5) | 1294 (2.3) |
| Blekinge | 42182 (27.7) | 14591 (9.6) | 3789 (2.5) |
| Skåne | 315075 (24.8) | 107627 (8.5) | 28647 (2.3) |
| Halland | 78532 (25.4) | 26595 (8.6) | 6903 (2.2) |
| Västra Götaland | 372796 (23.1) | 123267 (7.6) | 31077 (1.9) |
| Värmland | 74260 (27.5) | 26227 (9.7) | 6917 (2.6) |
| Örebro | 70872 (24.9) | 23167 (8.1) | 5661 (2) |
| Västmanland | 66960 (25.9) | 23110 (8.9) | 6089 (2.4) |
| Dalarna | 76817 (27.9) | 26562 (9.7) | 6844 (2.5) |
| Gävleborg | 74605 (27.1) | 26090 (9.5) | 6887 (2.5) |
| Västernorrland | 65020 (27.2) | 22388 (9.4) | 5592 (2.3) |
| Jämtland | 32160 (25.8) | 10936 (8.8) | 2848 (2.3) |
| Västerbotten | 63816 (24.7) | 21842 (8.5) | 5601 (2.2) |
| Norrbotten | 66993 (27.3) | 23852 (9.7) | 6652 (2.7) |

*On the 1^st^ of January 2016

**Appendix 10.** Burden and prevalence of at least one, two, and three prognostic factors for severe COVID-19 in each Swedish county for a 10-years look back period*

| County | Prognostic factors, n (%) | | |
| --- | --- | --- | --- |
|  | **At least one** | **At least two** | **At least three** |
| Stockholm | 538895 (24.8) | 177094 (8.1) | 51705 (2.4) |
| Uppsala | 87734 (25.3) | 30516 (8.8) | 8903 (2.6) |
| Södermanland | 80895 (29.2) | 29432 (10.6) | 8528 (3.1) |
| Östergötland | 120727 (27.7) | 44450 (10.2) | 13091 (3) |
| Jönköping | 95013 (28) | 36184 (10.7) | 11012 (3.2) |
| Kronoberg | 54755 (29.4) | 19431 (10.4) | 5623 (3) |
| Kalmar | 72338 (31.2) | 28346 (12.2) | 8524 (3.7) |
| Gotland | 17955 (31.7) | 6581 (11.6) | 1786 (3.2) |
| Blekinge | 47593 (31.3) | 17904 (11.8) | 5145 (3.4) |
| Skåne | 359175 (28.2) | 131539 (10.3) | 39420 (3.1) |
| Halland | 89709 (29.1) | 33030 (10.7) | 9721 (3.2) |
| Västra Götaland | 423649 (26.3) | 152483 (9.5) | 43520 (2.7) |
| Värmland | 81127 (30) | 31129 (11.5) | 9108 (3.4) |
| Örebro | 78872 (27.7) | 28524 (10) | 7895 (2.8) |
| Västmanland | 75572 (29.2) | 28215 (10.9) | 8235 (3.2) |
| Dalarna | 87394 (31.8) | 32493 (11.8) | 9229 (3.4) |
| Gävleborg | 83453 (30.3) | 31476 (11.4) | 9038 (3.3) |
| Västernorrland | 72630 (30.4) | 27252 (11.4) | 7545 (3.2) |
| Jämtland | 35824 (28.7) | 13236 (10.6) | 3785 (3) |
| Västerbotten | 73201 (28.3) | 27081 (10.5) | 7862 (3) |
| Norrbotten | 76090 (31) | 29487 (12) | 8984 (3.7) |

*On the 1^st^ of January 2016

**Appendix 11.** Burden and prevalence of underlying medical conditions suggesting high risk for severe COVID-19 by age group, with restricted cardiovascular and cancer code lists*

| Age groups  (years) | Cardiovascular disease  n (%) | Cancer  n (%) | COPD  n (%) | Severe asthma  n (%) | Diabetes  n (%) |
| --- | --- | --- | --- | --- | --- |
| 1-9 | 1756 (0.2) | 412 (0) | 652 (0.1) | 70549 (6.8) | 1817 (0.2) |
| 10-19 | 2502 (0.2) | 294 (0) | 330 (0) | 39110 (3.7) | 7192 (0.7) |
| 20-29 | 5743 (0.4) | 1182 (0.1) | 266 (0) | 15132 (1.2) | 13264 (1) |
| 30-39 | 6742 (0.6) | 2994 (0.2) | 371 (0) | 12633 (1.1) | 18407 (1.5) |
| 40-49 | 15721 (1.2) | 7264 (0.6) | 1525 (0.1) | 15266 (1.2) | 34611 (2.7) |
| 50-59 | 39066 (3.2) | 14901 (1.2) | 6429 (0.5) | 16281 (1.3) | 70427 (5.8) |
| 60-69 | 87011 (7.6) | 33034 (2.9) | 18923 (1.7) | 17521 (1.5) | 121901 (10.6) |
| 70-79 | 127742 (14.9) | 36259 (4.2) | 29321 (3.4) | 16794 (2) | 129137 (15.1) |
| 80+ | 132868 (26.5) | 18682 (3.7) | 20699 (4.1) | 12507 (2.5) | 74259 (14.8) |

*On the 1^st^ of January 2016

| County | Cardiovascular disease  n (%) | Cancer  n (%) | COPD  n (%) | Severe asthma  n (%) | Diabetes  n (%) |
| --- | --- | --- | --- | --- | --- |
| Stockholm | 75407 (3.5) | 24316 (1.1) | 16729 (0.8) | 56251 (2.6) | 86195 (4) |
| Uppsala | 12801 (3.7) | 3945 (1.1) | 2615 (0.8) | 8193 (2.4) | 16311 (4.7) |
| Södermanland | 12167 (4.4) | 3215 (1.2) | 2923 (1.1) | 5861 (2.1) | 15119 (5.5) |
| Östergötland | 19891 (4.6) | 5553 (1.3) | 3766 (0.9) | 9807 (2.2) | 21929 (5) |
| Jönköping | 16899 (5) | 4179 (1.2) | 3295 (1) | 8121 (2.4) | 18028 (5.3) |
| Kronoberg | 7729 (4.1) | 2311 (1.2) | 1704 (0.9) | 4608 (2.5) | 9272 (5) |
| Kalmar | 12793 (5.5) | 3192 (1.4) | 2448 (1.1) | 4782 (2.1) | 13347 (5.8) |
| Gotland | 2734 (4.8) | 765 (1.4) | 549 (1) | 1219 (2.2) | 3022 (5.3) |
| Blekinge | 7097 (4.7) | 2094 (1.4) | 1435 (0.9) | 3257 (2.1) | 8080 (5.3) |
| Skåne | 58577 (4.6) | 15690 (1.2) | 11791 (0.9) | 27964 (2.2) | 64259 (5.1) |
| Halland | 13732 (4.5) | 4130 (1.3) | 2276 (0.7) | 7300 (2.4) | 13653 (4.4) |
| Västra Götaland | 66032 (4.1) | 18958 (1.2) | 11545 (0.7) | 29353 (1.8) | 75352 (4.7) |
| Värmland | 14144 (5.2) | 3475 (1.3) | 2189 (0.8) | 6048 (2.2) | 16784 (6.2) |
| Örebro | 11397 (4) | 3122 (1.1) | 2039 (0.7) | 5243 (1.8) | 14831 (5.2) |
| Västmanland | 12614 (4.9) | 3262 (1.3) | 2244 (0.9) | 5446 (2.1) | 14240 (5.5) |
| Dalarna | 15519 (5.6) | 3222 (1.2) | 2133 (0.8) | 6512 (2.4) | 16283 (5.9) |
| Gävleborg | 14630 (5.3) | 3537 (1.3) | 2299 (0.8) | 6353 (2.3) | 16031 (5.8) |
| Västernorrland | 12469 (5.2) | 2955 (1.2) | 1694 (0.7) | 4764 (2) | 14545 (6.1) |
| Jämtland | 5649 (4.5) | 1602 (1.3) | 1019 (0.8) | 2485 (2) | 7154 (5.7) |
| Västerbotten | 12748 (4.9) | 2923 (1.1) | 1708 (0.7) | 5819 (2.3) | 12675 (4.9) |
| Norrbotten | 14122 (5.8) | 2576 (1) | 2115 (0.9) | 6407 (2.6) | 13905 (5.7) |

**Appendix 12.** Burden and prevalence of prognostic factors for severe COVID-19 by each Swedish county, with restricted cardiovascular and cancer code lists *

*On the 1^st^ of January 2016

| County | Prognostic factors, n (%) | | |
| --- | --- | --- | --- |
|  | **At least one** | **At least two** | **At least three** |
| Stockholm | 382293 (17.6) | 94386 (4.3) | 20993 (1) |
| Uppsala | 66535 (19.2) | 17055 (4.9) | 3917 (1.1) |
| Södermanland | 62422 (22.5) | 16395 (5.9) | 3729 (1.3) |
| Östergötland | 92634 (21.2) | 25028 (5.7) | 5719 (1.3) |
| Jönköping | 73681 (21.7) | 21491 (6.3) | 5274 (1.6) |
| Kronoberg | 41202 (22.1) | 10437 (5.6) | 2299 (1.2) |
| Kalmar | 56634 (24.4) | 16386 (7.1) | 3828 (1.7) |
| Gotland | 13668 (24.1) | 3518 (6.2) | 734 (1.3) |
| Blekinge | 35980 (23.6) | 9583 (6.3) | 2130 (1.4) |
| Skåne | 265280 (20.9) | 72420 (5.7) | 16454 (1.3) |
| Halland | 66652 (21.6) | 17296 (5.6) | 3753 (1.2) |
| Västra Götaland | 318528 (19.8) | 83612 (5.2) | 17985 (1.1) |
| Värmland | 65253 (24.2) | 18384 (6.8) | 4066 (1.5) |
| Örebro | 60628 (21.3) | 15406 (5.4) | 3159 (1.1) |
| Västmanland | 57717 (22.3) | 16082 (6.2) | 3737 (1.4) |
| Dalarna | 66625 (24.2) | 18755 (6.8) | 4132 (1.5) |
| Gävleborg | 65301 (23.7) | 18475 (6.7) | 4226 (1.5) |
| Västernorrland | 57295 (24) | 15728 (6.6) | 3319 (1.4) |
| Jämtland | 28551 (22.9) | 7752 (6.2) | 1710 (1.4) |
| Västerbotten | 55029 (21.3) | 15081 (5.8) | 3393 (1.3) |
| Norrbotten | 57983 (23.6) | 16800 (6.8) | 4038 (1.6) |

**Appendix 13.** Burden and prevalence of at least one, two, or three prognostic factors for severe COVID-19 in each Swedish county, with restricted cardiovascular and cancer code lists *

*On the 1^st^ of January 2016
